# Genome-wide RNA-sequencing dataset reveals *AC096751.1* sever as a novel prognostic long non-coding RNA and its potential molecular mechanisms in patients with colon adenocarcinoma

**DOI:** 10.7150/jca.83424

**Published:** 2023-07-31

**Authors:** Cun Liao, Zhiwen Gu, Wei Huang, Yizhen Gong, Xiwen Liao, Minglin Lin, Sen Zhang

**Affiliations:** 1 Department of Colorectal and Anal Surgery, The First Affiliated Hospital of Guangxi Medical University, Nanning, 530021, Guangxi Zhuang Autonomous Region, People's Republic of China.; 2 Guangxi Key Laboratory of Enhanced Recovery after Surgery for Gastrointestinal Cancer, Nanning, 530021, Guangxi Zhuang Autonomous Region, People's Republic of China.; 3 Department of Clinical Research, Guangxi Medical University Cancer Hospital, 530021, Guangxi Zhuang Autonomous Region, People's Republic of China.; 4 Department of Hepatobiliary Surgery, The First Affiliated Hospital of Guangxi Medical University, Nanning, 530021, Guangxi Zhuang Autonomous Region, People's Republic of China.

**Keywords:** long non-coding RNA, *AC096751.1*, colon adenocarcinoma, overall survival, The Cancer Genome Atlas.

## Abstract

**Objective**: Through data analysis, we observed that *AC096751.1* is markedly imbalance between colon adenocarcinoma (COAD) cancer and paracancerous tissues. However, the prognostic value and potential molecular mechanism of *AC096751.1* in COAD are still unclear.

**Methods:** Whole genome RNA-sequencing datasets of The Cancer Genome Atlas (TCGA) COAD cohort were collected into current study, comprehensive survival analysis and bioinformatics function enrichment analysis approaches were apply to explore the clinical outcome and molecular mechanisms of *AC096751.1* in COAD.

**Results:** In current study, we found that AC096751.1 is markedly down-regulated in COAD cancer tissues (log2 fold change =2.303, *P*<0.0001, false discovery rate <0.0001), and can be serve as a biomarker to distinguish COAD cancer and paracancerous tissues [area under curve=0.9518, 95% confidence interval (CI)=0.9261-0.9776]. Survival analysis suggests that low expression of AC096751.1 is connected with poor clinical outcome of COAD, and can serve as a novel prognostic indicator (log-rank P=0.016, adjusted P=0.005, hazard ratio=0.548, 95% CI=0.360-0.836). Bioinformatics function enrichment analysis suggests that the molecular mechanism of AC096751.1 in COAD may include participation in cell adhesion, cell proliferation, mitogen-activated protein kinase kinase (MAPKK), MAPK, janus-activated kinase-singal transducers and activators of transcriprion cascade, Erk1 and Erk 2 cascade, and nuclear factor-kappa B pathway. Tumor microenvironment and immune infiltration analysis indicates that COAD patients with different AC096751.1 expression have significant variation in tumor immune background.

**Conclusion:** The present study found that *AC096751.1* is significantly differentially expressed in COAD and can be serve as a novel prognostic biomarker.

## Introduction

Colon cancer is a common malignancy of digestive tract 1, 2. Colon cancer has a high mortality rate, and if it can be detected and treated early, it can effectively improve the survival rate of colon cancer patients. Therefore, early diagnosis and treatment can markedly affect the prognosis of colon cancer patients 1, 2. Colon adenocarcinoma (COAD) is the most common pathological type of colon cancer. Studies show that non-coding RNAs play a crucial role in epigenetics. It is important in the growth and differentiation of cells and the occurrence and development of cancers 3-6. With the rapid development of high-throughput sequencing, more and more novel cancer-related lncRNAs have been discovered. By downloading The Cancer Genome Atlas (TCGA) COAD cohort RNA sequencing data set, we found that *AC096751.1* was closely related to COAD OS through genome-wide prognostic lncRNA screening minning (note: the data screening process is not shown in current study), and was significantly differentially expressed between cancer and paracancerous tissues. Through literature search, we did not find any reports about AC096751.1 in previous studies. This means that we have discovered a novel lncRNA, which is dysregulated in COAD cancer tissues and is closely related to COAD OS. To fully investigate the clinical significance and potential molecular mechanism of AC096751.1 in COAD, we performed this study. The main objective of our study was to comprehensively investigate the prognostic value and molecular mechanisms of *AC096751.1* in COAD using the whole genome RNA-sequencing dataset from TCGA cohort.

## Materials and methods

### COAD dataset acquisition

The original COAD dataset was got from the TCGA website (https://portal.gdc.cancer.gov/), including clinical and RNA sequencing datasets 7. RNA sequencing data normalization is performed by *edgeR* package 8, 9. In total, we obtained RNA sequencing data from 521 samples, including 41 non-tumor and 480 tumor tissues. By matching RNA sequencing data and clinical parameters, we included 438 patients with survival parameters and expression profile data for subsequent prognostic analysis. We collected 31 pairs tumor and para-carcinoma tissues from patients with COAD in the Department of Colorectal and Anal Surgery, the First Affiliated Hospital of Guangxi Medical University during January 1, 2020 to August 30, 2020. These patients did not receive any treatment before surgery, and the pathological diagnosis was COAD after surgical resection. This study was approved by the Ethics Committee of the First Affiliated Hospital of Guangxi Medical University, the approval number is 2021 (KY-E-182). The procedures for this study are in line with the Declaration of Helsinki and its subsequent modifications. Written informed consent was obtained from all participants.

### Reverse Transcription-Polymerase Chain Reaction (RT-PCR)

The primer sequence was synthesized by Beijing TSINGKE Biological Technology Co., Ltd. (Nanning). The primer sequence of *AC096751.1* was shown below: F: 5'-CCCCGTGATGCAGAGAACTT-3'; R: 5'-GCCCAGATAGCGTTCCTTGT-3'. NucleoZol RNA extraction reagent was used for RNA extraction from fresh tissues. CDNA reverse transcription was performed according to the Prime Script RT Reagent Kit with gDNA Eraser (TAKARA DRR047A) Kit. RT-PCR system is as follows: Fast Start Universal SYBR Green Master (ROX): 10ul; Primer F (10uM): 0.6ul; Primer R (10uM): 0.6ul; DdH2O: 6.4 ul; ROX Reference Dye II: 0.4ul; CDNA: 2 ul; and the total volume is 20ul. RT-PCR was then performed in Appliedbiosystems QuantStudio 6 Flex System. After obtaining RT-PCR results, the expression level of AC096751.1 was calculated according to 2^-△△^CT method.

### Survival analysis of *AC096751.1*

The scatter plot and receiver operating characteristic (ROC) curve of *AC096751.1* in COAD cancer and paracancer tissues are used to evaluate its diagnostic value. We conducted a comprehensive assessment of the prognostic value of *AC096751.1* in COAD using univariate and multivariate Cox proportional hazards regression model. The log-rank test of Kaplan-Meier method is also used for survival analysis. The nomogram and joint effect prognostic analysis are applied to assess the potential application value of *AC096751.1* and other clinical indicators in COAD OS.

### Functional enrichment analysis

Various approaches were applied to comprehensively analyze the molecular mechanisms of *AC096751.1* in COAD. We use the *Cor* function in R platform to perform genome-wide co-expression analysis of *AC096751.1* and protein-coding genes, and the co-expression correlation intensity is evaluated by Pearson's correlation coefficient (r). Prognostic analysis of co-expressed genes was performed in the R platform by *survival* package (https://cran.r-project.org/web/packages/survival/index.html). The gene ontology (GO) and Kyoto Encyclopedia of Genes and Genomes (KEGG) functional enrichment analysis of co-expressed genes was carried out by Database for Annotation, Visualization and Integrated Discovery v6.8 (DAVID, https://david.ncifcrf.gov/home.jsp) 10, 11. BinGO analysis was also used to verify DAVID's enrichment results 12. To further explore the molecular mechanisms of *AC096751.1* in COAD, we further analyzed the molecular mechanisms involved in the prognosis difference between high- and low-*AC096751.1* expression phenotypes in COAD by gene set enrichment analysis (GSEA, http://software.broadinstitute.org/gsea/index.jsp) 13, 14. The reference gene sets were selected from c2 (c2.all.v7.4.symbols.gmt) and c5 (c5.all.v7.4.symbols.gmt) gene sets derived from Molecular Signatures Database (MSigDB) 15, 16. The criteria of significance results for GSEA are as follows: |Normalized Enrichment Score (NES)| >1, false discovery rate (FDR)<0.25 and P <0.05. The molecular mechanism of *AC096751.1* was further explored by screening the differentially expressed genes (DEGs) between high- and low-*AC096751.1* expression phenotypes in COAD. DEGs screening were performed by *edgeR* package. DEGs were identified as log_2_|fold change (FC)|>1, P<0.05 and FDR<0.05. The functional enrichment analysis and survival analysis of DEGs were as described above. Subsequently, we used the connectivity map (CMap, https://portals.broadinstitute.org/cmap/) website to screen AC096751.1 targeted drugs in COAD based on the obtained DEGs 17.

### Tumor microenvironment and immune infiltration of *AC096751.1* in COAD

Tumor microenvironment scores are scored based on genome-wide RNA sequencing dataset using the Estimation of STromal and Immune cells in MAlignant Tumor tissues using Expression data (ESTIMATE) package 18. Finally, three scores of stromal, immune and ESTIMATE score in the tumor microenvironment are obtained. We use the single sample GSEA (ssGSEA) method to compare the differences of immune infiltration between high- and low-AC096751.1 expression groups in COAD, which were performed by GSVA in R platform 19.

### Statistical analysis

Independent sample t test is used for continuous variable data. The strength of co-expression correlation is evaluated using Pearson's correlation coefficient. FDR is carried out by the Benjamini-Hochberg procedure 20. Hazard ratio (HR) and 95% confidence interval (CI) are two indicators applied to assess the difference in prognosis. All statistical analysis adopts SPSS version 26.0 and R version 4.0.2. P<0.05 considered significant difference.

## Results

### Survival analysis of *AC096751.1*

**Figure 1:**
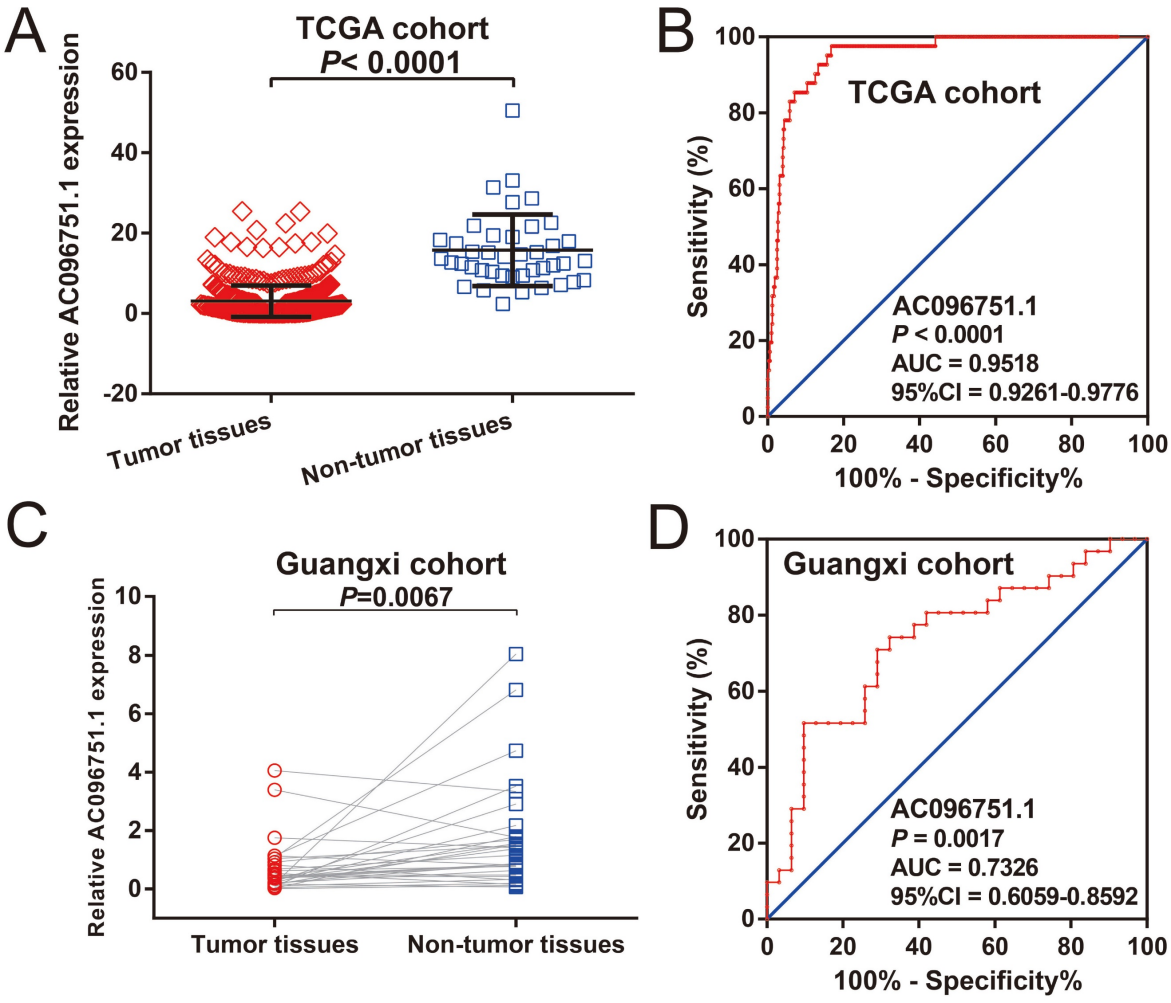
Expression distribution of *AC096751.1* between COAD carcinoma and adjacent tissues. (A) Scatter plot of *AC096751.1* expression in COAD carcinoma and adjacent tissues in TCGA cohort; (B) ROC curve of *AC096751.1* in distinguish COAD carcinoma and adjacent tissues in TCGA cohort. (C) Scatter plot of *AC096751.1* expression in COAD carcinoma and adjacent tissues in Guangxi cohort; (D) ROC curve of *AC096751.1* in distinguish COAD carcinoma and adjacent tissues in Guangxi cohort.

**Figure 2:**
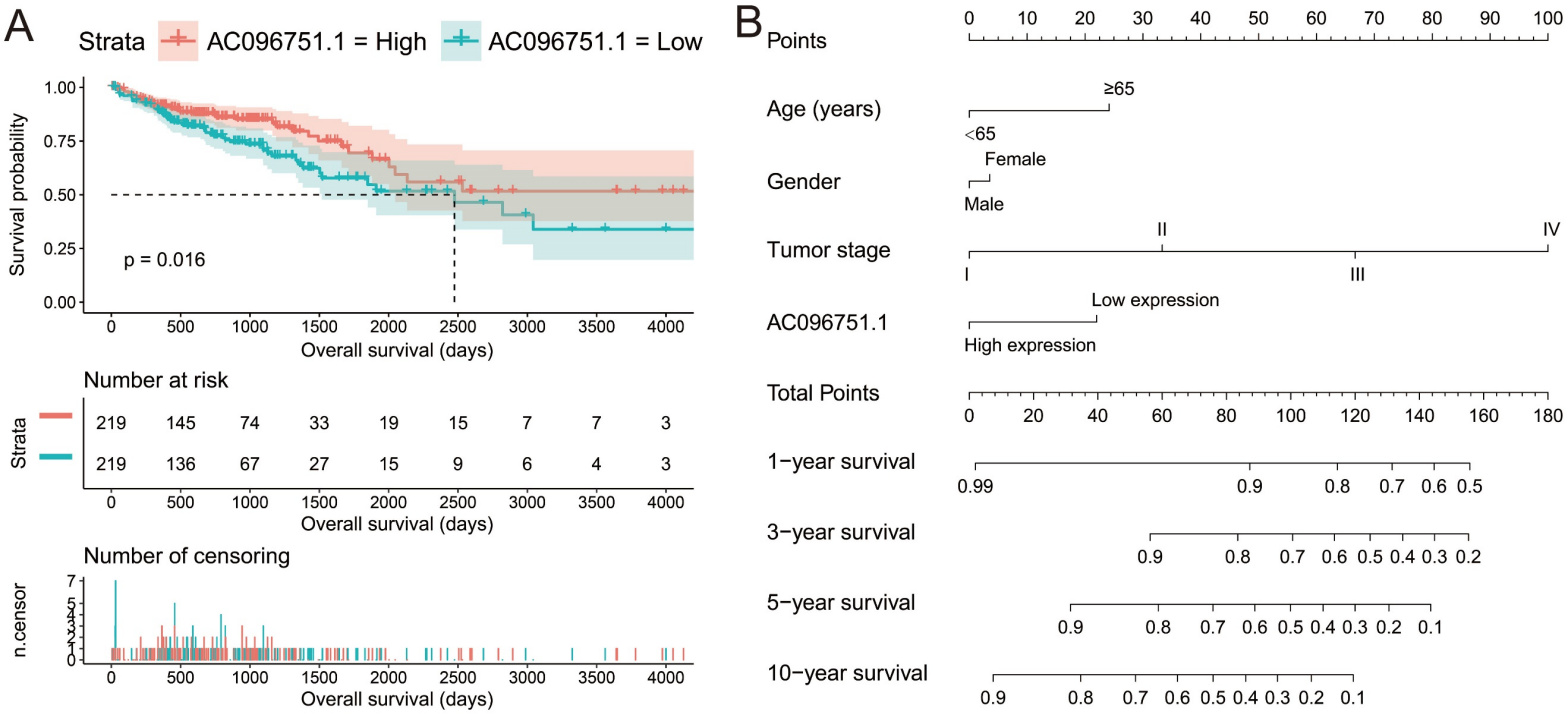
Prognostic value of *AC096751.1* in COAD. (A) Kaplan-Meier curve of *AC096751.1* in COAD; (B) Nomogram of *AC096751.1* in COAD.

**Figure 3:**
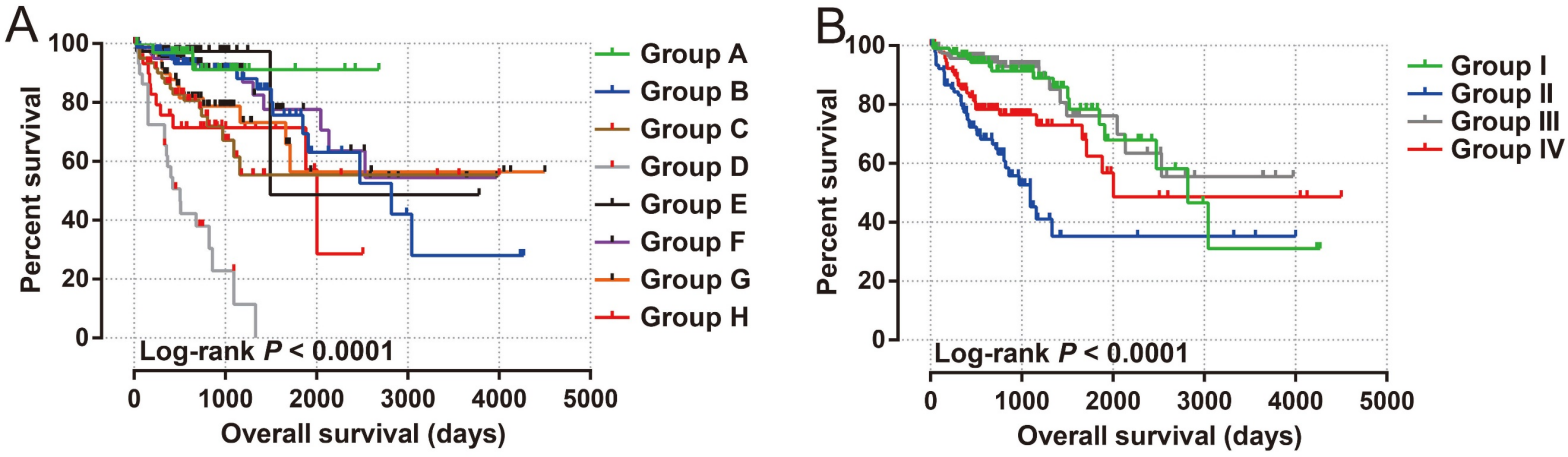
Joint effect survival analysis of *AC096751.1* in COAD. (A) *AC096751.1* combined with tumor stage (I, II, III and IV); (B) *AC096751.1* combined with tumor stage (I+II and III+IV).

The clinical indicators of COAD patients are showed in **Table S1**. The tumor stage is significantly related to COAD overall survival (OS), and the multivariate Cox proportional hazards regression model needs to be included for correction (**Table S1**). By comparing the expression distribution of *AC096751.1* in cancer and paracancerous tissues, we observed that *AC096751.1* was markedly down-regulated in COAD cancer tissues (**Figure 1A**, log_2_ FC=2.303, *P*<0.0001, FDR<0.0001). ROC analysis indicated that *AC096751.1* could distinguish COAD carcinoma from paracancerous tissues with high accuracy, and the area under curve (AUC) is 0.9518, the 95%CI is 0.9261 to 0.9776 (**Figure 1B**, *P*<0.0001). In the Guangxi cohort, we also observed that AC096751.1 was significantly up-regulated in paracancerous tissues (P=0.0067, **Figure 1C**). ROC also suggests that AC096751.1 has a higher diagnostic value (P=0.0017, AUC=0.7326, 95%CI=0.6059-0.8592, **Figure 1D**). Survival analysis revealed that COAD patients with low *AC096751.1* expression had a significantly increased risk of death than patients with high *AC096751.1* expression, and suggesting a poor prognosis (log-rank *P*=0.016, adjusted *P*=0.005, HR=0.548, 95%CI=0.360-0.836, **Figure 2A**). The nomogram indicates that the tumor stage is dominant in the contribution of COAD prognosis, and the contribution of *AC096751.1* expression to COAD OS ranks third (**Figure 2B**). Then we combined *AC096751.1* with tumor stage for joint effect survival analysis, we have observed that combining these two indicators for survival analysis can classify COAD patients more accurately. Through this classification, COAD with different prognosis can be accurately distinguished (**Figure 3A-B**, **Table 1**).

### Functional enrichment analysis

**Figure 4:**
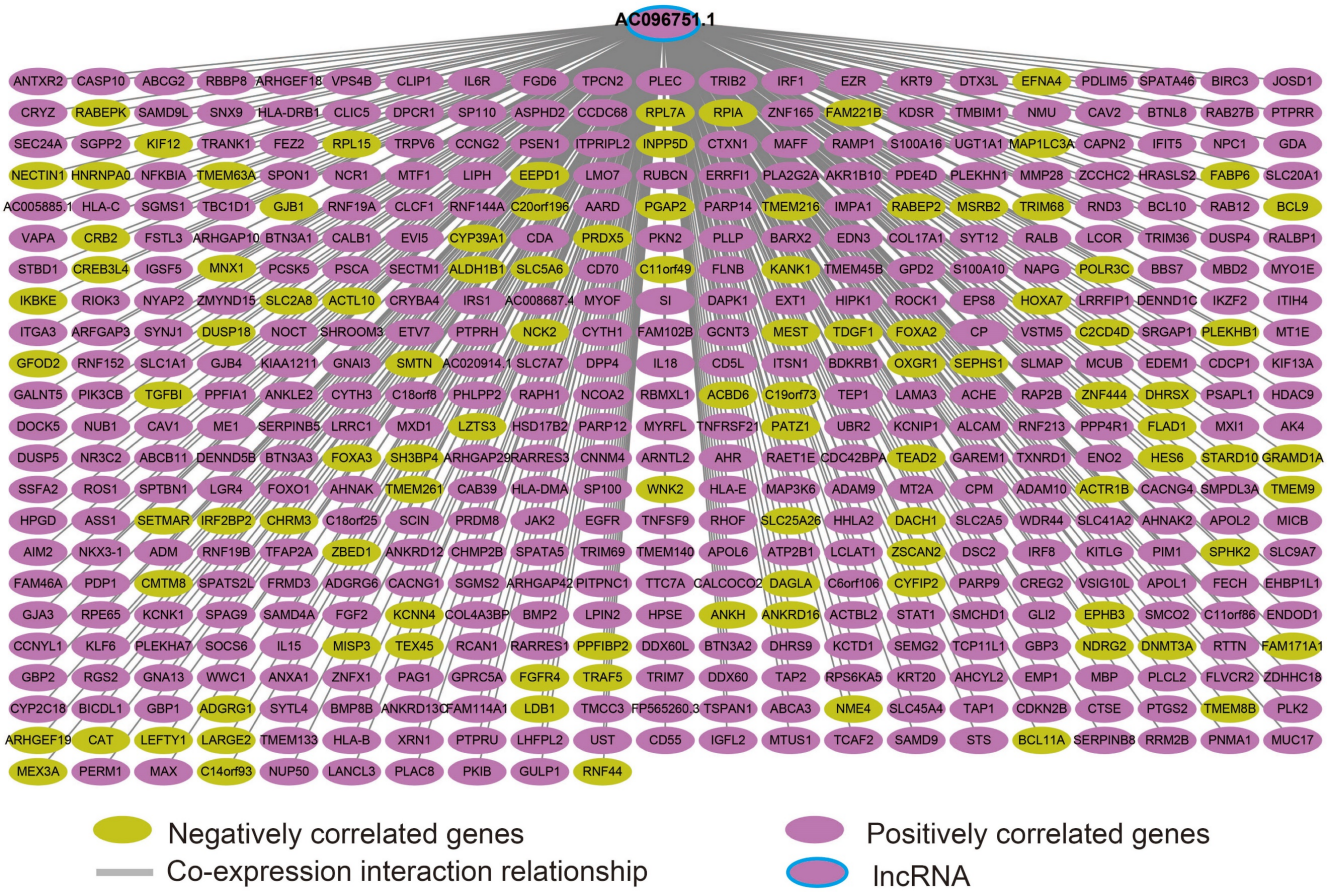
Co-expression interaction network of *AC096751.1* in COAD.

**Figure 5:**
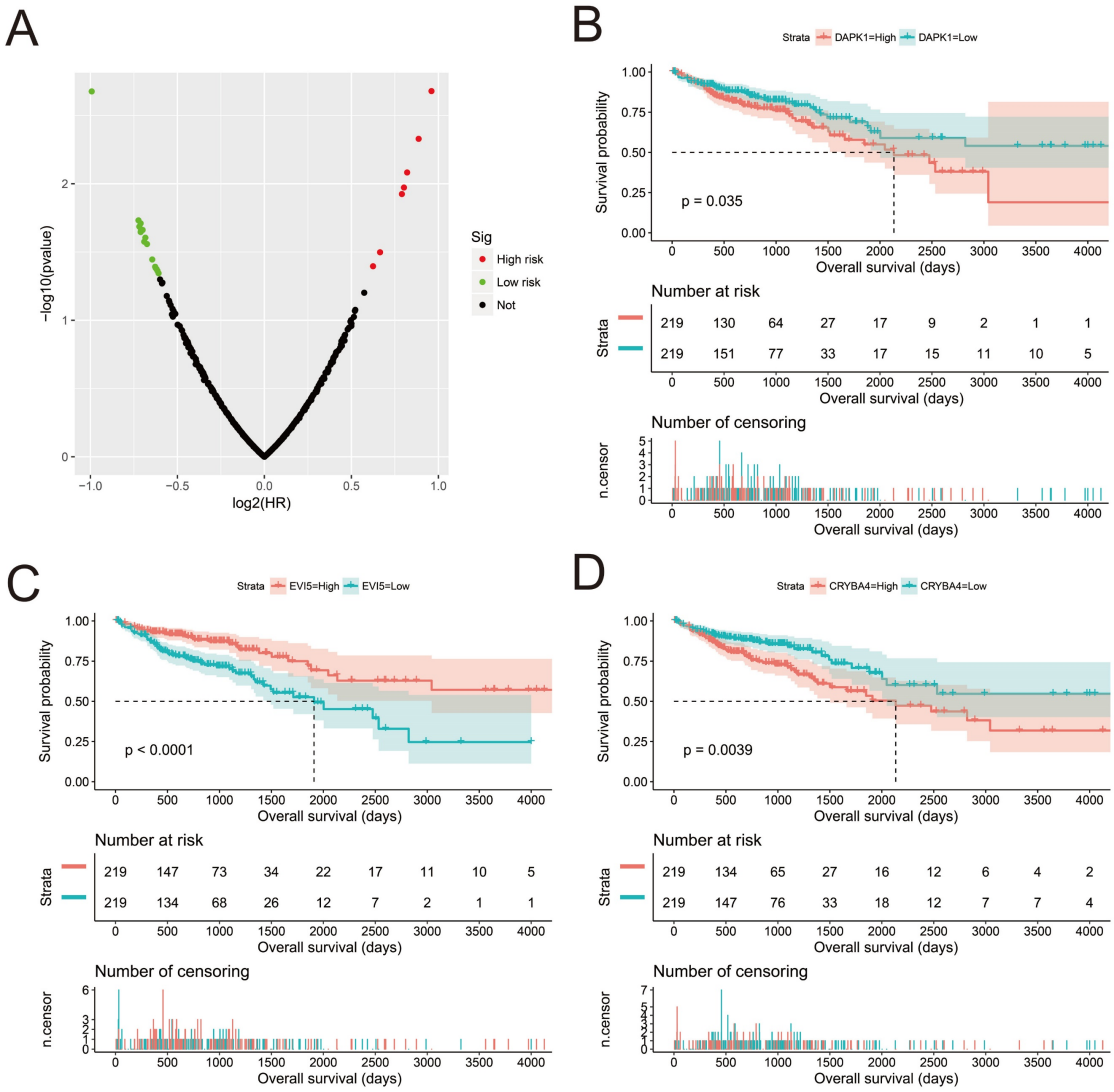
Survival analysis of *AC096751.1* co-expressed genes in COAD OS. (A) Volcano plot of survival analysis results of *AC096751.1* co-expressed genes; (B) Kaplan-Meier curve of DAPK1; (C) Kaplan-Meier curve of EVI5; (D) Kaplan-Meier curve of CRYBA4.

**Figure 6:**
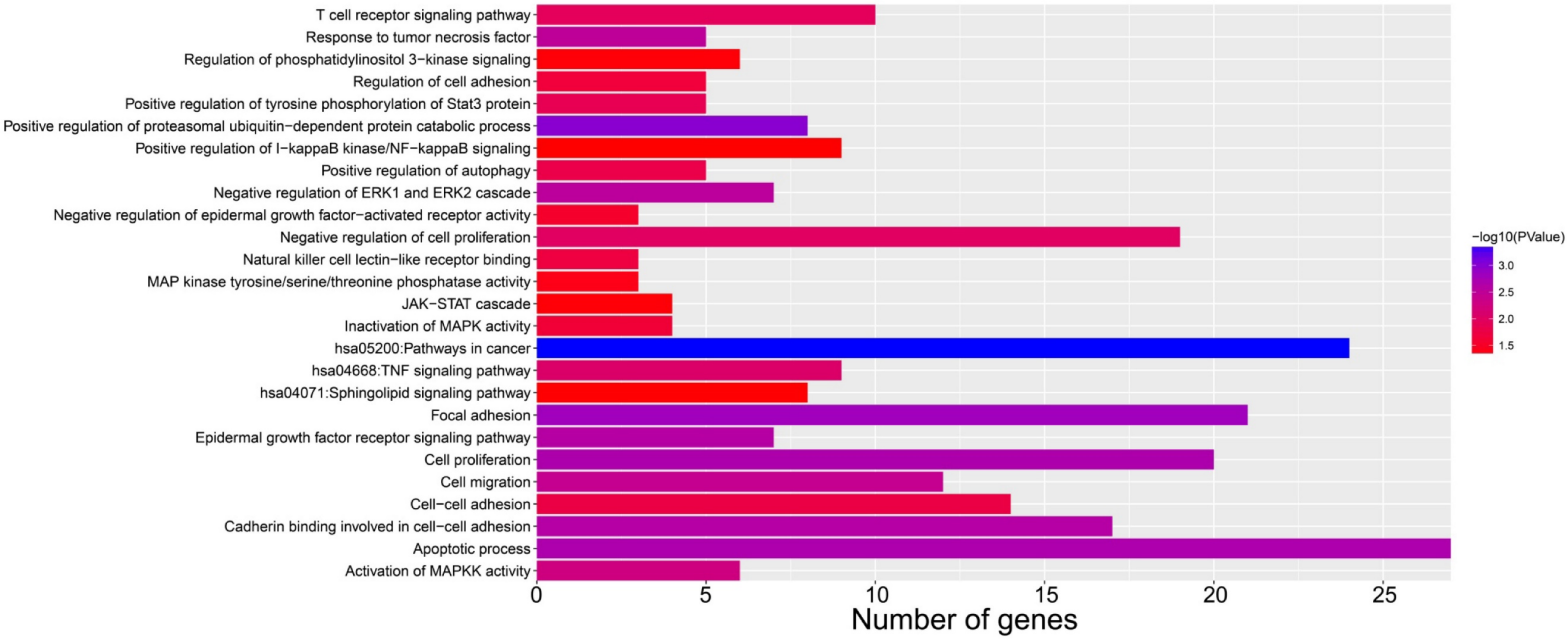
Bar plot of *AC096751.1* co-expressed gene functional enrichment analysis results.

We screened the co-expressed genes of *AC096751.1* in COAD cancer tissues to further understand its potential molecular mechanisms in COAD. A total of 472 co-expressed genes of *AC096751.1* were obtained, including 372 positively correlated protein coding genes and 100 negatively correlated protein coding genes (**Table S2 and Figure 4**). Through the survival analysis of these co-expressed genes, we obtained a total of 23 genes closely related to COAD OS (**Table S3 and Figure 5A**), including 7 high risk genes (HR>1) and 16 low risk genes (HR<1). Among them, the three most significant genes are death associated protein kinase 1 (DAPK1), ecotropic viral integration site 5 (EVI5) and crystallin beta A4 (CRYBA4) in order (**Figure 5B-D**). GO term analysis indicated that these co-expressed genes were closely related to the following biological processes: positive regulation of proteasomal ubiquitin-dependent protein catabolic process, focal adhesion, apoptotic process, cell proliferation, cadherin binding involved in cell-cell adhesion, epidermal growth factor receptor signaling pathway, negative regulation of Erk1 and Erk 2 cascade, response to tumor necrosis factor, cell migration, activation of mitogen-activated protein kinase kinase (MAPKK) activity, negative regulation of cell proliferation, T cell receptor signaling pathway, positive regulation of tyrosine phosphorylation of Stat3 protein, positive regulation of autophagy, natural killer cell lectin-like receptor binding, janus-activated kinase-singal transducers and activators of transcriprion (JAK-STAT) cascade, regulation of phosphatidylinositol 3-kinase signaling, and positive regulation of I-kappaB kinase/ nuclear factor (NF)-kappa B signaling (**Table S4** and** Figure 6**). KEGG analysis indicated that these co-expressed genes were closely related to the following pathways: pathways in cancer, tumor necrosis factor (TNF) signaling pathway, and sphingolipid signaling pathway (**Table S4** and** Figure 6**). BinGO analysis also partially supports our above functional enrichment analysis results, which are significantly enriched to the following biological processes: regulation of cell migration, regulation of signaling pathway, regulation of cell proliferation, positive regulation of cell migration, regulation of cell adhesion, positive regulation of phosphorylation, activation of MAPKK activity, negative regulation of apoptosis (**Figure S1**).

**Figure 7:**
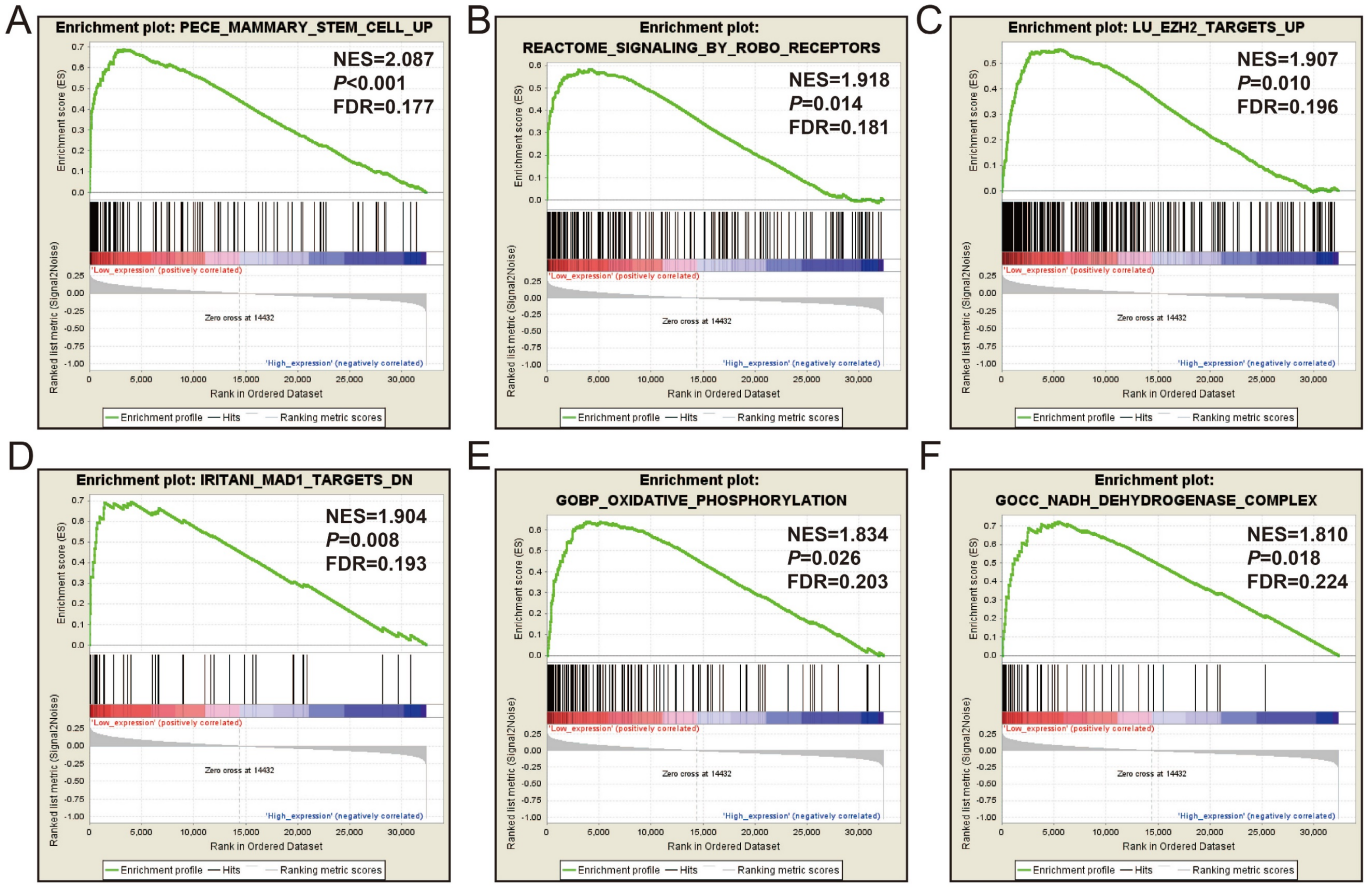
GSEA results between high- and low-*AC096751.1* expression phenotypes in COAD. (A) mammary stem cell up; (B) signaling by Robo receptors; (C) EZH2 targets up; (D) MAD1 targets dn; (E) oxidative phosphorylation; (F) NADH dehydrogenase complex.

**Figure 8:**
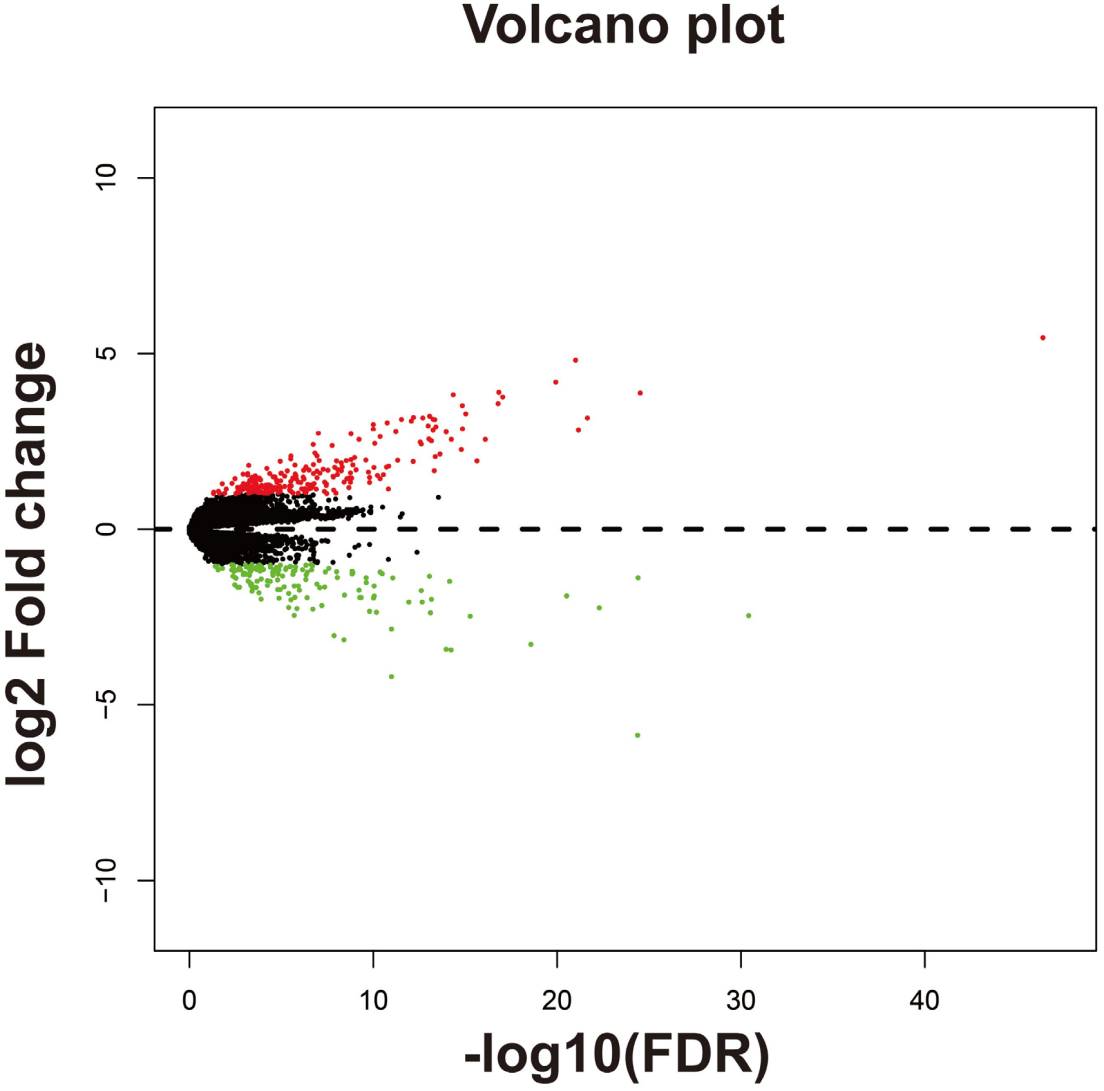
Volcano plot of DEGs between high- and low-*AC096751.1* expression phenotypes.

**Figure 9:**
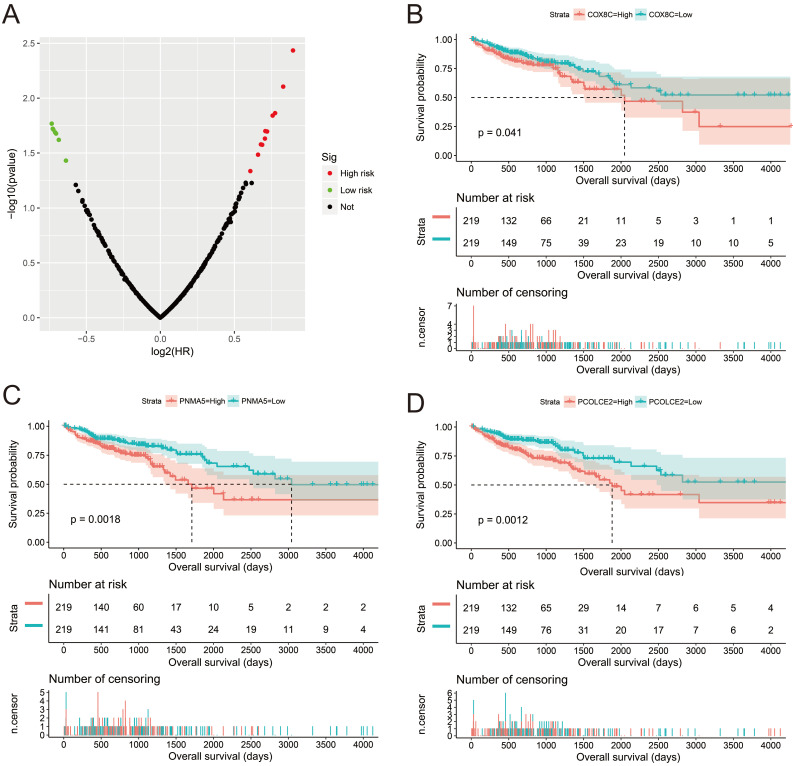
Survival analysis of DEGs between high- and low-*AC096751.1* expression phenotypes in COAD OS. (A) Volcano plot of survival analysis results of DEGs; (B) Kaplan-Meier curve of COX8C; (C) Kaplan-Meier curve of PNMA5; (D) Kaplan-Meier curve of PCOLCE2.

**Figure 10:**
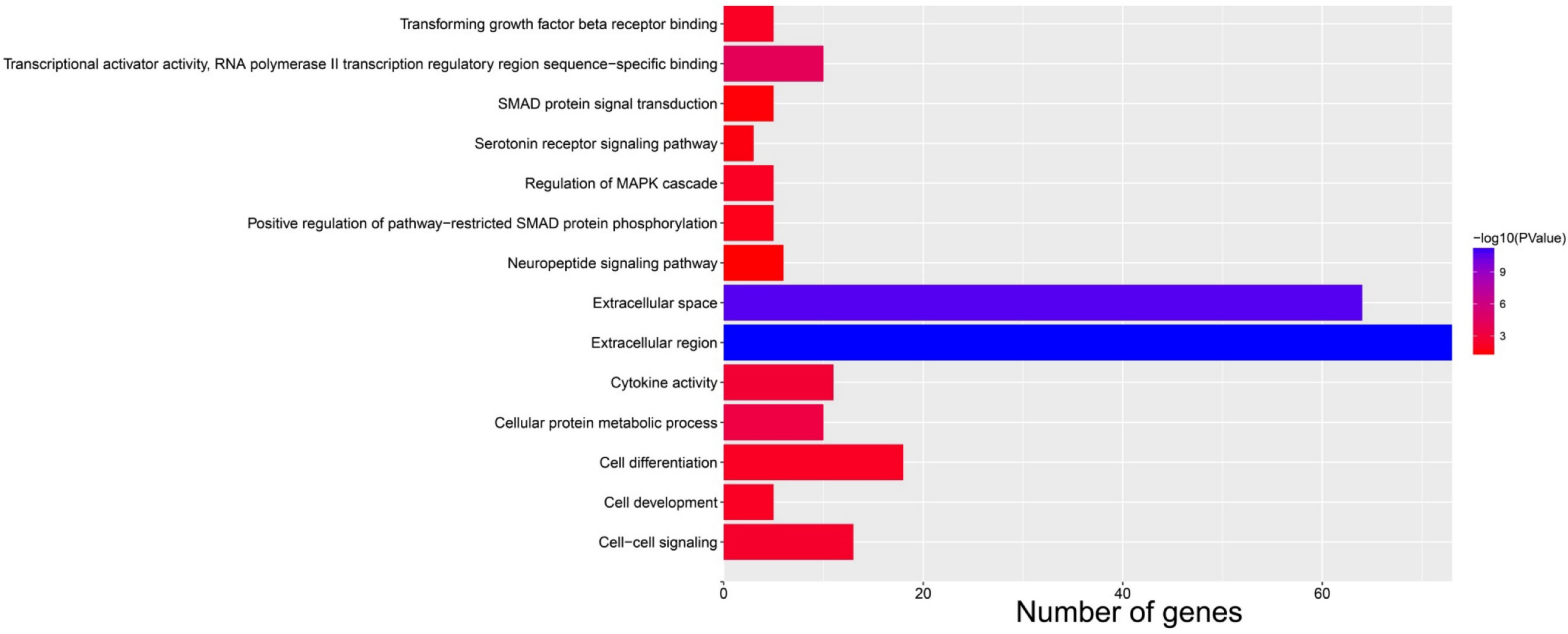
Bar plot for functional enrichment analysis results of DEGs between high- and low-*AC096751.1* expression phenotypes.

Subsequently, we used GSEA analysis to investigate the molecular mechanism of *AC096751.1*. The following molecular mechanisms can be significantly enriched by GSEA, including: mammary stem cell up, signaling by Robo receptors, enhancer of zeste 2 polycomb repressive complex 2 subunit (EZH2) targets up, mitotic arrest deficient 1 like 1 (MAD1) targets dn, oxidative phosphorylation, nicotinamide adenine dinucleotide (NADH) dehydrogenase complex (**Figure 7A-F** and **Table S5**). A total of 375 genes were screened out and differentially expressed in COAD tissues between high- and low-*AC096751.1* expression phenotypes (**Figure 8**, **Figure S2** and **Table S6**). Among them, 146 DEGs were markedly down-regulated and 229 DEGs were markedly up-regulated. Through the survival analysis of these DEGs, we got a total of 18 DEGs closely related to COAD OS(**Table S7 and Figure 9A**), including eleven high risk DEGs (HR>1) and seven low risk DEGs (HR<1). Among them, the three most significant genes are cytochrome C oxidase subunit 8C (COX8C,** Figure 9B**), PNMA family member 5 (PNMA5,** Figure 9C**), procollagen C-endopeptidase enhancer 2 (PCOLCE2,** Figure 9D**). GO term analysis indicated that these co-expressed genes were closely related to the following biological processes: cytokine activity, cell-cell signaling, regulation of MAPK cascade, transforming growth factor beta receptor binding, cell differentiation, positive regulation of pathway-restricted SMAD protein phosphorylation, serotonin receptor signaling pathway, SMAD protein signal transduction, neuropeptide signaling pathway (**Figure 10** and **Table S8**). KEGG analysis indicated that these DEGs were closely related to the following pathways: neuroactive ligand-receptor interaction, protein digestion and absorption, alcoholism and pancreatic secretion (**Table S8**). BinGO analysis also partially supports our above functional enrichment analysis results, which are significantly enriched to the following biological processes: cell differentiation, cell-cell signaling, growth factor activity and cytokine activity (**Figure S3**). Based on the filtered DEGs, we also screened three targeted drugs (MK-886, quipazine and lovastatin) of AC096751.1 in COAD by CMap (**Table 2**).

### Tumor microenvironment and immune infiltration of *AC096751.1* in COAD

**Figure 11:**
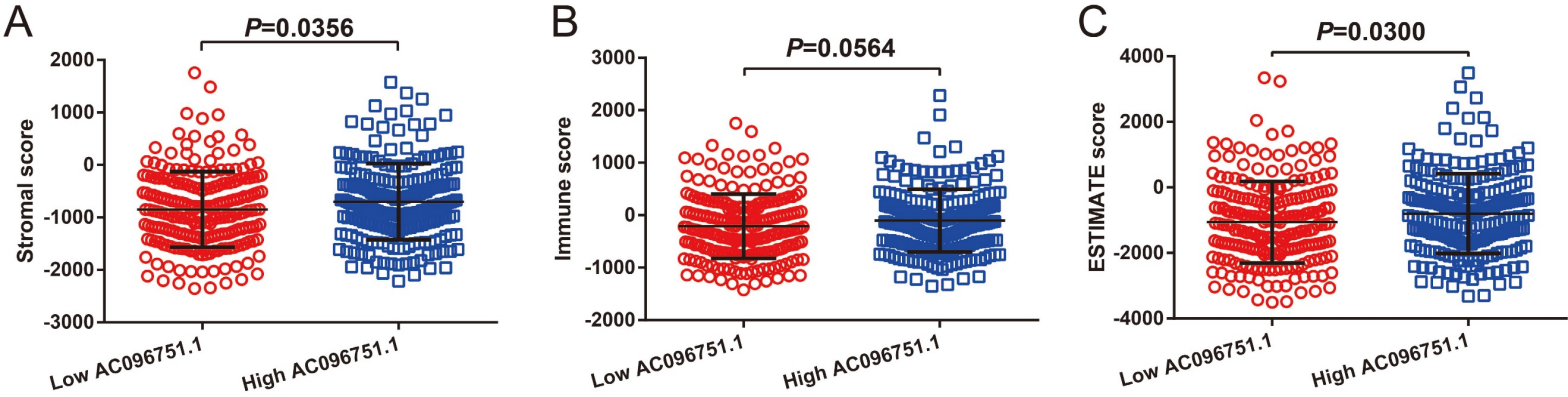
The relationship between *AC096751.1* and tumor microenvironment score in COAD. (A) Scatter plot of stromal score and *AC096751.1*; (B) Scatter plot of immune score and *AC096751.1*; (C) Scatter plot of ESTIMATE score and *AC096751.1*.

**Figure 12:**
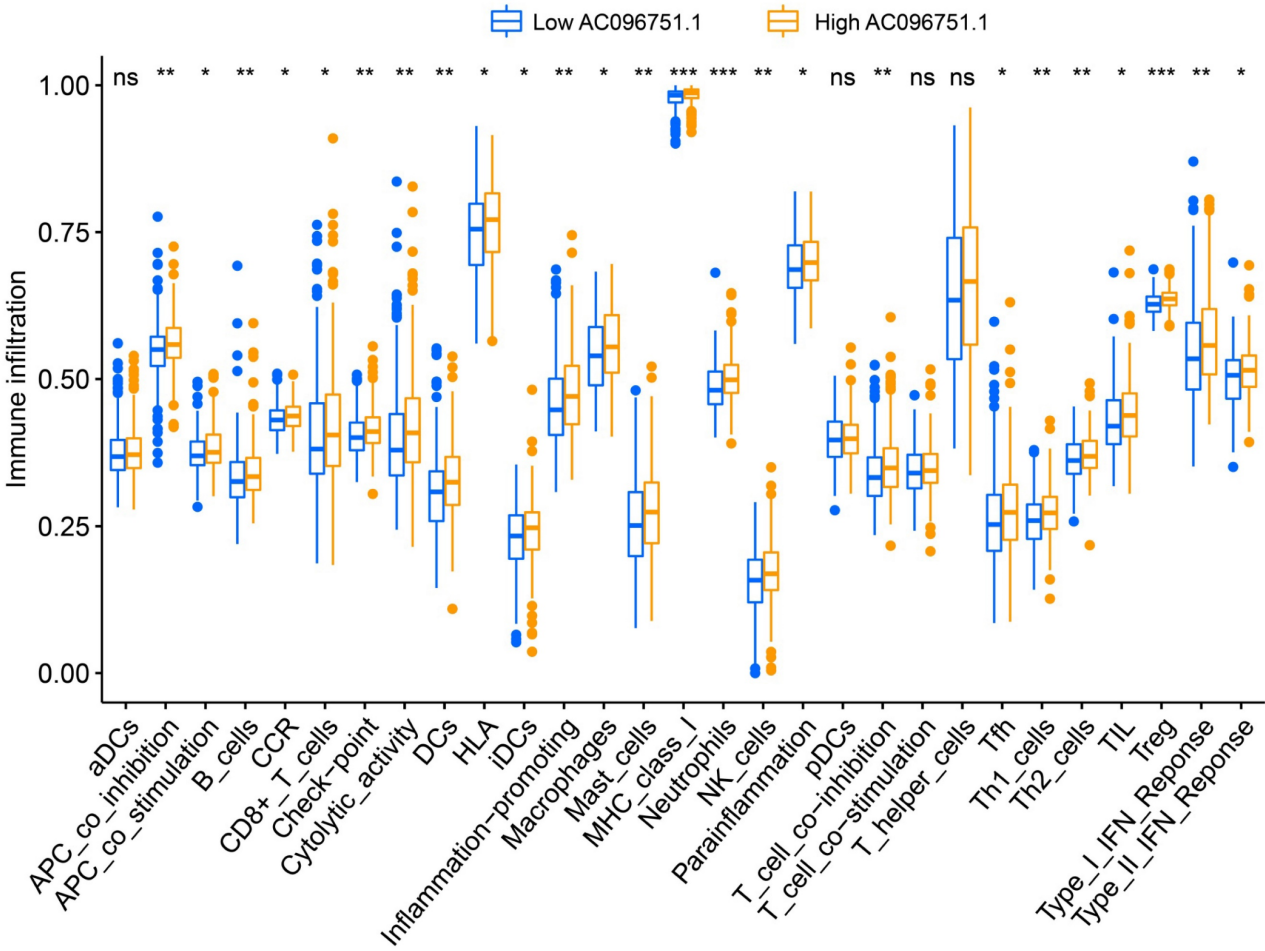
Immune cell infiltration difference box plot between high- and low-AC096751.1 expression phenotypes in COAD. **Notes**: *P<0.05, **P<0.01, ***P<0.001.

Through tumor microenvironment analysis, we obtained the scores of three microenvironment indicators in cancer tissues of COAD patients. Through comparison, we observe that there are significant differences in stromal scores between COAD patients with high- and low-*AC096751.1* expression phenotypes. The stromal score of high-AC096751.1 COAD patients was markedly higher than that of those with low expression (*P*=0.0356, **Figure 11A**). There was no significant difference in immune score between the two groups COAD patients (*P*=0.0564, **Figure 11B**). The ESTIMATE score of high-AC096751.1 COAD patients was markedly higher than that of those with low expression (*P*=0.0300, **Figure 11C**). By comparing the differences in immune cell infiltration between high- and low-AC096751.1 phenotypes, we found that most immune cells are significantly different between the two groups. We found that the abundance of immune cell infiltration was significantly higher in COAD patients with high-AC096751.1 than in patients with low-AC096751.1 (**Figure 12**).

## Discussion

In recent years, more and more studies have been conducted on lncRNAs-related analysis based on the TCGA COAD cohort, and a number of studies have conducted comprehensive screening and identification of prognostic lncRNAs in the TCGA COAD cohort. Zhang et al. screened the differentially expressed RNAs in cancer and paracancerous tissues by RNA sequencing and miRNA sequencing in the TCGA COAD cohort and constructed the ceRNA regulatory network 21. Mao et al. constructed competing endogenous RNA (cRNAs) from the RNA sequencing data set of GSE39582 and TCGA COAD, and screened out the lncRNAs related to COAD recurrence 22. Xing et al. also developed a 14-lncRNA signature using RNA sequencing data from the TCGA COAD cohort to predict survival in patients with COAD 23. Zeng et al developed a four-lncRNA signature for predicting the survival of colorectal adenocarcinoma patients through a similar approach, including COAD and rectal adenocarcinoma 24. Although several studies have screened prognostic lncRNAs in the TCGA COAD cohort, the prognostic value of AC096751.1 in COAD OS has not been reported in these studies. The main reason may be that these studies were the screening of genome-wide lncRNAs, which mainly focused on the lncRNAs with high significance, and did not pay much attention to these novel lncRNAs that did not rank very high in significance. The present study is the first comprehensive investigation of the clinical significance and potential molecular mechanisms of AC096751.1 in COAD OS. Compared with previous studies, our current study found a novel lncRNA that differentially expressed and prognostic related in COAD.

Through the prognostic analysis of the co-expressed genes and differentially expressed genes of AC096751.1, we found that the prognostic genes screened were also closely related to cancers in previous studies. DAPK1 has a higher promoter hypermethylation frequency in colorectal carcinogenesis, which may be a genetic marker of colorectal carcinogenesis 25. Xu et al. reported in previous studies that DAPK1 is closely related to the prognosis of COAD 26. Liu et al. found that DAPK1 was dysregulated in COAD cancer tissue by analyzing the differentially expressed genes between cancer and paracancerous tissues, and could be used as a diagnostic marker for early COAD 27. Luo et al. 's study found that grifolin can induce the expression of DAPK1 in nasopharyngeal cancer, breast cancer and colon cancer cell lines, thereby targeting the regulation of P53 protein to induce apoptosis of cancer cells. This study suggests that DAPK1 can be used as a target for tumor targeted therapy 28. Study has shown that pyrimidine-based DAPK1/colony stimulating factor 1 receptor (CSF1R) dual inhibitor can significantly inhibit the proliferation of various tumor cell lines, including colon cancer cell line^29^. Xie et al. showed that DAPK1 expression level was negatively correlated with tumor stage through expression profile data set, and low DAPK1 expression was markedly correlated with poor prognosis of bladder cancer. DAPK1 is an important prognostic marker and therapeutic target for bladder cancer, while vemurafenib and trimetinib may be potential targeting drugs for DAPK1 in bladder cancer 30. Li et al. detected the expression level of DAPK1 in liver cancer tissues by immunohistochemical method and found that it was significantly down-regulated in liver cancer tissues, and low expression of DAPK1 was associated with poor prognosis of liver cancer. Bioinformatics suggests that amcinonide and sulpiride may be potential targeting agents for DAPK1 in liver cancer 31. Song et al. analyzed the TCGA data set and found that low DAPK1 is associated with poor prognosis in patients with clear cell renal cell carcinoma (ccRCC). In vivo and in vitro experiments confirmed that DAPK1 is associated with sunitinib resistance in ccRCC 32. Previous studies have found that the DAPK1 gene is abnormally methylated in a variety of tumors, including brain metastases, squamous cell carcinoma, gliomas, high-grade cervical lesions, breast cancer, myelodysplastic syndrome, gastric cancer and non-small cell lung cancer (NSCLC) 33-40. The CpG site of DAPK1 is also related to the prognosis of glioma patients 36. DAPK1 can also play an oncogene effect in p53-mutated cancers, and high expression of DAPK1 can promote the growth of p53-mutated tumors 41. Chou et al. evaluated the five-year survival of 99 breast cancer patients and found that DAPK1 was not significantly associated with the prognosis of breast cancer 42. Li et al. confirmed through cell experiments that miR-135b can promote tumor cell invasion and metastasis by regulating EVI5 in hepatocellular carcinoma (HCC) 43. In addition, study has found that EVI5 can be serve as a novel prognostic biomarker for HCC 44. EVi5 has been shown to function as an oncogene in multiple cancers, including NSCLC, HCC and laryngeal cancer 43, 45, 46. Study by Zeng et al. suggests that CRYBA4 was differentially expressed in ccRCC tissues and was significantly correlated with prognosis 47.

Phelan et al. observed that COX8C was significantly overexpressed in patients with Barrett's esophagus and dysplastic, which may have some clinical value in the diagnosis of this disease 48. PNMA5 plays an oncogene effect in human breast cancer and cervical cancer cell lines, can promote cancer cell apoptosis and enhance chemosensitivity 49. Research by Huang et al. showed that PNMA5 plays an oncogene role in NSCLC and can promote the occurrence of bone metastasis 50. Through genome-wide differential expression gene screening, Wang et al. observed that PNMA5 was significantly differentially expressed in glioblastoma cancer tissues, and it was closely related to the prognosis of glioblastoma 51. The prognosis of glioblastoma patients with high expression of PNMA5 was poor 51. Based on the TCGA colon cancer cohort, Zhou et al. found that PNMA5 is significantly differentially expressed in colon cancer, and high expression of PNMA5 is associated with poor prognosis of colon cancer. They also constructed a prognostic signature containing PNMA5, which can divide colon cancer into two subtypes with significant differences in prognosis 52. Chen et al. analyzed the TCGA colorectal cancer cohort and found that patients with high expression of PCOLCE2 have a poor prognosis. In addition, they also constructed a nine-gene prognostic signature that includes PCOLCE2 to accurately predict the survival of colorectal cancer patients 53. Zhang et al. 's study revealed that patients with uterine corpus endometrial carcinoma with high expression of PCOLCE2 have a poor prognosis 54. PCOLCE2 gene methylation plays the role of hub gene in nasopharyngeal carcinoma 55. In this study, we used different bioinformatics functional enrichment analysis methods to reveal that the molecular mechanisms of AC096751.1 in COAD may involve a variety of classic tumor-related signaling pathways, including MAPK, MAPKK, JAK-STAT and NF-kappa B pathways. These signaling pathways and biological processes are closely related to the occurrence, development and prognosis of cancers.

This study has certain shortcomings. First of all, this study is a single-cohort study and lacks an additional validation cohort. Due to the limited clinical parameters provided by TCGA, we could not obtain as many clinical parameters of patients as possible for multivariate analysis. Secondly, because AC096751.1 is a newly discovered COAD prognosis-related lncRNA marker, it has not been reported in the past. There is no relevant research support for its molecular mechanism in tumors. The molecular mechanism obtained through bioinformatics analysis in this study, and lacks in vivo and in vitro experiments verification. Despite the above deficiencies, our study is the first to report the potential clinical significance and molecular mechanism of AC096751.1 in COAD. It has certain application value in translational medicine. It can provide data support and study direction for the subsequent exploration of the molecular mechanism of the clinical significance of AC096751.1 in cancers.

## Conclusions

In conclusion, our results suggest that AC096751.1 is markedly down-regulated in COAD cancer tissues, and can serve as a biomarker to distinguish COAD cancer and paracancerous tissues. Survival analysis also suggests that low expression of AC096751.1 is closely related to poor prognosis of COAD, and can serve as a novel prognostic biomarker. Functional enrichment analysis suggests that the molecular mechanism of AC096751.1 in COAD may include participation in cell adhesion, cell proliferation, MAPK, MAPKK, JAK-STAT cascade, Erk1 and Erk 2 cascade, and NF-kappa B pathway. We also screened three targeted drugs (MK-886, quipazine and lovastatin) of AC096751.1 in COAD by CMap. Tumor microenvironment analysis indicates that there are significant differences in stromal and ESTIMATE scores between COAD patients with different AC096751.1 expression levels. The abundance of immune cell infiltration in tumor tissues of COAD patients with high-AC096751.1 were significantly increased. This phenomenon suggests that differences in tumor microenvironment may have a certain impact on the prognosis of COAD patients. However, our findings still need to be further proved in future studies.

## Supplementary Material

Supplementary figures and tables.

## Figures and Tables

**Table 1:** Joint effects survival analysis of tumor stage and the *AC096751.1* expression with OS in COAD patients

Group	*AC096751.1*	Tumor stage†	Patients (n=427)₰	MST (days)	Crude HR (95% CI)	Crude *P*	Adjusted HR (95% CI)	Adjusted *P* £
								
**A**	**Low expression**	**I**	35	NA	1		1	
**B**	**Low expression**	**II**	85	2821	2.464(0.562-10.800)	0.232	2.464(0.562-10.800)	0.232
**C**	**Low expression**	**III**	63	NA	4.944(1.140-21.433)	0.033	4.944(1.140-21.433)	0.033
**D**	**Low expression**	**IV**	31	504	22.103(5.151-94.840)	<0.001	22.103(5.151-94.840)	<0.001
**E**	**High expression**	**I**	38	1493	1.072(0.151-7.621)	0.945	1.072(0.151-7.621)	0.945
**F**	**High expression**	**II**	82	NA	2.021(0.452-9.044)	0.357	2.021(0.452-9.044)	0.357
**G**	**High expression**	**III**	63	NA	3.488(0.792-15.369)	0.099	3.488(0.792-15.369)	0.099
**H**	**High expression**	**IV**	30	2003	5.800(1.268-26.522)	0.023	5.800(1.268-26.522)	0.023
								
**I**	**Low expression**	**I+II**	120	2821	1		1	
**II**	**Low expression**	**III+IV**	94	1094	4.057(2.276-7.231)	<0.001	16.249(5.336-49.481)	<0.001
**III**	**High expression**	**I+II**	120	NA	0.853(0.420-1.732)	0.660	0.847(0.417-1.720)	0.646
**IV**	**High expression**	**III+IV**	93	2003	1.984(1.066-3.695)	0.031	7.106(2.303-21.926)	0.001

**Notes**: £Adjusted for tumor stage. † Tumor stage information are unavailable in 11 patients. ₰ Due to the lack of tumor stage information in 11 patients, only 427 of 438 patients were included in the combined survival analysis. **Abbreviation:** OS, overall survival; COAD, colon adenocarcinoma; MST, median survival time; HR, hazard ratio; CI, confidence interval.

**Table 2:** CMap analysis results

Name	Mean connective score	n	Enrichment	P value	Specificity	Percent non-null
MK-886	-0.51	2	-0.963	0.00304	<0.01	100
Quipazine	-0.384	4	-0.709	0.01484	0.0063	50
Lovastatin	-0.406	4	-0.708	0.01486	0.0155	50

**Abbreviation:** CMap: connectivity map.
