# Supplementary material for: Genome-wide RNA-sequencing dataset reveals AC096751.1 sever as a novel prognostic long non-coding RNA and its potential molecular mechanisms in patients with colon adenocarcinoma
Source: J Cancer. 2023 Jul 31;14(12):2386–98. doi: 10.7150/jca.83424 (PMC10414039; doi:10.7150/jca.83424)
Supplement: Supplementary file 1 — Supplementary figures and tables. [file jcav14p2386s1.pdf]

Table S1. Clinical parameters of TCGA COAD cohort

Variables	Patients (n=438)	MST(days)	HR(95%CI)	Log-rank P
**Gender**				0.545
**Female**	204	NA	1	
**Male**	234	2475	1.131(0.759-1.686)	
**Age (years) §**				0.112
**<65**	168	NA	1	
**≥65**	268	2475	1.420(0.919-2.194)	
**Tumor stage £**				<0.0001
**I**	73	NA	1	
**II**	167	2821	2.240(0.781-6.421)	
**III**	126	NA	4.068(1.434-11.538)	
**IV**	61	858	11.291(3.980-32.026)	
**Tumor stage**				<0.0001
**I+II**	240	3042	1	
**III+IV**	187	1711	3.097(2.009-4.772)	
**AC096751.1**				0.016
**Low expression**	219	2475	1	
**High expression**	219	NA	0.610(0.407-0.915)	

Notes: §Information of age was unavailable in 2 patients; £Information of tumor stage was unavailable in 11 patients.

Abbreviation: NA,Not available; TCGA, The Cancer Genome Atlas; MST, median survival time;COAD, colon adenocarcinoma; HR, hazard ratio; CI, confidence interval.

Table S2. List of co-expressed genes of AC096751.1

ID	ID	r	pValue
**AC096751.1**	AC096751.1	1	9.07E-30
**AC096751.1**	HPGD	0.621	2.32E-12
**AC096751.1**	FLVCR2	0.376	2.42E-05
**AC096751.1**	BIRC3	0.361	5.10E-05
**AC096751.1**	PIM1	0.349	9.07E-05
**AC096751.1**	DENND5B	0.346	0.000104
**AC096751.1**	PLAC8	0.344	0.000115
**AC096751.1**	GCNT3	0.344	0.000115
**AC096751.1**	CCDC68	0.336	0.000166
**AC096751.1**	HSD17B2	0.331	0.000208
**AC096751.1**	RARRES3	0.328	0.000238
**AC096751.1**	PARP12	0.324	0.000284
**AC096751.1**	PTPRR	0.319	0.000354
**AC096751.1**	IRS1	0.318	0.00037
**AC096751.1**	GRAMD1A	-0.305	0.000403
**AC096751.1**	DAPK1	0.314	0.000439
**AC096751.1**	SOCS6	0.312	0.000479
**AC096751.1**	RND3	0.312	0.000479
**AC096751.1**	FAM46A	0.311	0.000499
**AC096751.1**	KCNK1	0.31	0.000521
**AC096751.1**	KCTD1	0.31	0.000521
**AC096751.1**	KLF6	0.31	0.000521
**AC096751.1**	PKIB	0.309	0.000544
**AC096751.1**	EGFR	0.308	0.000567
**AC096751.1**	ENDOD1	0.308	0.000567
**AC096751.1**	IMPA1	0.305	0.000643
**AC096751.1**	SAMD9	0.304	0.000671
**AC096751.1**	PAG1	0.3	0.000791
**AC096751.1**	TMEM133	0.3	0.000791
**AC096751.1**	GPD2	0.298	0.000859
**AC096751.1**	RNF213	0.297	0.000895
**AC096751.1**	PTGS2	0.296	0.000932
**AC096751.1**	TEAD2	-0.284	0.00097
**AC096751.1**	MXD1	0.295	0.000971
**AC096751.1**	APOL2	0.294	0.001011
**AC096751.1**	RIOK3	0.294	0.001011
**AC096751.1**	DUSP5	0.293	0.001053
**AC096751.1**	SEMG2	0.292	0.001096
**AC096751.1**	EMP1	0.292	0.001096
**AC096751.1**	EZR	0.291	0.001141
**AC096751.1**	ARFGAP3	0.291	0.001141
**AC096751.1**	DPCR1	0.29	0.001188

**AC096751.1**	ARHGAP10	0.288	0.001286
**AC096751.1**	SETMAR	-0.276	0.001337
**AC096751.1**	PPP4R1	0.287	0.001338
**AC096751.1**	WDR44	0.287	0.001338
**AC096751.1**	SMCHD1	0.287	0.001338
**AC096751.1**	TDGF1	-0.275	0.001391
**AC096751.1**	SAMD9L	0.286	0.001392
**AC096751.1**	CALCOCO2	0.286	0.001392
**AC096751.1**	ARHGAP42	0.285	0.001448
**AC096751.1**	DPP4	0.285	0.001448
**AC096751.1**	NYAP2	0.284	0.001506
**AC096751.1**	CACNG4	0.284	0.001506
**AC096751.1**	WWC1	0.283	0.001567
**AC096751.1**	TMCC3	0.283	0.001567
**AC096751.1**	PNMA1	0.282	0.001629
**AC096751.1**	HPSE	0.282	0.001629
**AC096751.1**	SCIN	0.281	0.001694
**AC096751.1**	TCAF2	0.281	0.001694
**AC096751.1**	KDSR	0.28	0.001761
**AC096751.1**	PCSK5	0.279	0.00183
**AC096751.1**	CDKN2B	0.279	0.00183
**AC096751.1**	SLC41A2	0.279	0.00183
**AC096751.1**	EFNA4	-0.267	0.001901
**AC096751.1**	OXGR1	-0.267	0.001901
**AC096751.1**	ADAM9	0.277	0.001977
**AC096751.1**	ARHGAP29	0.277	0.001977
**AC096751.1**	IGFL2	0.277	0.001977
**AC096751.1**	C18orf8	0.276	0.002054
**AC096751.1**	ALDH1B1	-0.264	0.002133
**AC096751.1**	EXT1	0.275	0.002134
**AC096751.1**	TCP11L1	0.274	0.002217
**AC096751.1**	CREG2	0.272	0.002392
**AC096751.1**	DOCK5	0.272	0.002392
**AC096751.1**	PHLPP2	0.272	0.002392
**AC096751.1**	AHNAK	0.272	0.002392
**AC096751.1**	CPM	0.272	0.002392
**AC096751.1**	LRRC1	0.272	0.002392
**AC096751.1**	GBP3	0.271	0.002484
**AC096751.1**	TEX45	-0.259	0.002577
**AC096751.1**	PSAPL1	0.27	0.002579
**AC096751.1**	SHROOM3	0.27	0.002579
**AC096751.1**	PARP14	0.267	0.002885
**AC096751.1**	RNF44	-0.255	0.002992
**AC096751.1**	CASP10	0.266	0.002994

**AC096751.1**	STS	0.266	0.002994
**AC096751.1**	PKN2	0.266	0.002994
**AC096751.1**	ABCB11	0.266	0.002994
**AC096751.1**	CYTH3	0.265	0.003107
**AC096751.1**	NUB1	0.265	0.003107
**AC096751.1**	IL15	0.265	0.003107
**AC096751.1**	MBD2	0.265	0.003107
**AC096751.1**	PSCA	0.264	0.003224
**AC096751.1**	MYOF	0.264	0.003224
**AC096751.1**	SYTL4	0.264	0.003224
**AC096751.1**	WNK2	-0.252	0.003343
**AC096751.1**	FOXO1	0.263	0.003345
**AC096751.1**	KIAA1211	0.263	0.003345
**AC096751.1**	PTPRH	0.262	0.003469
**AC096751.1**	NPC1	0.262	0.003469
**AC096751.1**	JOSD1	0.262	0.003469
**AC096751.1**	RAB27B	0.26	0.003732
**AC096751.1**	GDA	0.26	0.003732
**AC096751.1**	GBP2	0.259	0.00387
**AC096751.1**	DACH1	-0.247	0.00401
**AC096751.1**	SPON1	0.258	0.004013
**AC096751.1**	IKZF2	0.258	0.004013
**AC096751.1**	CP	0.257	0.00416
**AC096751.1**	RNF152	0.257	0.00416
**AC096751.1**	VPS4B	0.257	0.00416
**AC096751.1**	LGR4	0.257	0.00416
**AC096751.1**	KIF13A	0.257	0.00416
**AC096751.1**	TMEM9	-0.245	0.00431
**AC096751.1**	KITLG	0.256	0.004312
**AC096751.1**	XRN1	0.256	0.004312
**AC096751.1**	VSTM5	0.256	0.004312
**AC096751.1**	VAPA	0.256	0.004312
**AC096751.1**	TTC7A	0.256	0.004312
**AC096751.1**	SLC1A1	0.255	0.004469
**AC096751.1**	CD5L	0.254	0.004632
**AC096751.1**	MT2A	0.254	0.004632
**AC096751.1**	GJB4	0.254	0.004632
**AC096751.1**	TRIM69	0.254	0.004632
**AC096751.1**	RRM2B	0.254	0.004632
**AC096751.1**	PLA2G2A	0.254	0.004632
**AC096751.1**	TMEM261	-0.242	0.004797
**AC096751.1**	NECTIN1	-0.242	0.004797
**AC096751.1**	CCNYL1	0.253	0.004799
**AC096751.1**	BTN3A3	0.253	0.004799

**AC096751.1**	HOXA7	-0.241	0.00497
**AC096751.1**	HNRNPA0	-0.241	0.00497
**AC096751.1**	SERPINB5	0.252	0.004973
**AC096751.1**	EPHB3	-0.24	0.005149
**AC096751.1**	LARGE2	-0.24	0.005149
**AC096751.1**	ANXA1	0.251	0.005151
**AC096751.1**	BTN3A1	0.251	0.005151
**AC096751.1**	IL18	0.251	0.005151
**AC096751.1**	NFKBIA	0.251	0.005151
**AC096751.1**	DHRS9	0.251	0.005151
**AC096751.1**	CRB2	-0.239	0.005333
**AC096751.1**	DDX60L	0.25	0.005336
**AC096751.1**	TAP1	0.25	0.005336
**AC096751.1**	SPTBN1	0.25	0.005336
**AC096751.1**	MBP	0.25	0.005336
**AC096751.1**	MEST	-0.238	0.005524
**AC096751.1**	GJB1	-0.238	0.005524
**AC096751.1**	KRT9	0.249	0.005527
**AC096751.1**	PDLIM5	0.249	0.005527
**AC096751.1**	NOCT	0.249	0.005527
**AC096751.1**	TMEM8B	-0.237	0.00572
**AC096751.1**	TRAF5	-0.237	0.00572
**AC096751.1**	HLA-DRB1	0.248	0.005723
**AC096751.1**	AC005885.1	0.248	0.005723
**AC096751.1**	COL17A1	0.248	0.005723
**AC096751.1**	BCL11A	-0.236	0.005923
**AC096751.1**	MISP3	-0.236	0.005923
**AC096751.1**	ADGRG6	0.247	0.005926
**AC096751.1**	DHRSX	-0.235	0.006132
**AC096751.1**	CACNG1	0.246	0.006136
**AC096751.1**	GNA13	0.246	0.006136
**AC096751.1**	LHFPL2	0.246	0.006136
**AC096751.1**	HLA-DMA	0.245	0.006352
**AC096751.1**	SYT12	0.245	0.006352
**AC096751.1**	FRMD3	0.244	0.006575
**AC096751.1**	NUP50	0.244	0.006575
**AC096751.1**	PPFIBP2	-0.232	0.006801
**AC096751.1**	LZTS3	-0.232	0.006801
**AC096751.1**	VSIG10L	0.243	0.006805
**AC096751.1**	GBP1	0.243	0.006805
**AC096751.1**	SP100	0.243	0.006805
**AC096751.1**	PRDM8	0.243	0.006805
**AC096751.1**	SH3BP4	-0.231	0.007038
**AC096751.1**	APOL6	0.242	0.007042

**AC096751.1**	FABP6	-0.23	0.007283
**AC096751.1**	CLIC5	0.241	0.007287
**AC096751.1**	MYO1E	0.241	0.007287
**AC096751.1**	CD70	0.241	0.007287
**AC096751.1**	DUSP4	0.241	0.007287
**AC096751.1**	SLC20A1	0.241	0.007287
**AC096751.1**	CYFIP2	-0.229	0.007535
**AC096751.1**	FOXA2	-0.229	0.007535
**AC096751.1**	ZBED1	-0.229	0.007535
**AC096751.1**	KANK1	-0.229	0.007535
**AC096751.1**	CAV2	0.24	0.007539
**AC096751.1**	FGD6	0.24	0.007539
**AC096751.1**	ROS1	0.24	0.007539
**AC096751.1**	GPRC5A	0.24	0.007539
**AC096751.1**	ITSN1	0.239	0.007799
**AC096751.1**	BTN3A2	0.239	0.007799
**AC096751.1**	CMTM8	-0.227	0.008062
**AC096751.1**	DDX60	0.238	0.008067
**AC096751.1**	CTXN1	0.238	0.008067
**AC096751.1**	PPFIA1	0.238	0.008067
**AC096751.1**	ACTR1B	-0.226	0.008338
**AC096751.1**	HES6	-0.226	0.008338
**AC096751.1**	C2CD4D	-0.226	0.008338
**AC096751.1**	GALNT5	0.237	0.008343
**AC096751.1**	KRT20	0.237	0.008343
**AC096751.1**	SRGAP1	0.236	0.008627
**AC096751.1**	PITPNC1	0.236	0.008627
**AC096751.1**	DNMT3A	-0.224	0.008916
**AC096751.1**	ADGRG1	-0.224	0.008916
**AC096751.1**	DAGLA	-0.224	0.008916
**AC096751.1**	IKBKE	-0.223	0.009218
**AC096751.1**	SAMD4A	0.234	0.009223
**AC096751.1**	UST	0.234	0.009223
**AC096751.1**	TMEM45B	0.234	0.009223
**AC096751.1**	TAP2	0.234	0.009223
**AC096751.1**	RTTN	0.234	0.009223
**AC096751.1**	CLIP1	0.234	0.009223
**AC096751.1**	ADAM10	0.234	0.009223
**AC096751.1**	PLEKHB1	-0.222	0.009529
**AC096751.1**	ITGA3	0.233	0.009534
**AC096751.1**	TNFSF9	0.233	0.009534
**AC096751.1**	CD55	0.233	0.009534
**AC096751.1**	CYP39A1	-0.221	0.009849
**AC096751.1**	CAPN2	0.232	0.009854

**AC096751.1**	STBD1	0.232	0.009854
**AC096751.1**	TGFBI	-0.22	0.010179
**AC096751.1**	TRIB2	0.231	0.010184
**AC096751.1**	TXNRD1	0.231	0.010184
**AC096751.1**	TRIM7	0.231	0.010184
**AC096751.1**	FLNB	0.231	0.010184
**AC096751.1**	AIM2	0.231	0.010184
**AC096751.1**	TRPV6	0.231	0.010184
**AC096751.1**	SPHK2	-0.219	0.010519
**AC096751.1**	RGS2	0.23	0.010524
**AC096751.1**	ANKRD12	0.23	0.010524
**AC096751.1**	IRF1	0.23	0.010524
**AC096751.1**	HDAC9	0.23	0.010524
**AC096751.1**	APOL1	0.23	0.010524
**AC096751.1**	ANKRD16	-0.218	0.010869
**AC096751.1**	NME4	-0.218	0.010869
**AC096751.1**	FGF2	0.229	0.010874
**AC096751.1**	C18orf25	0.229	0.010874
**AC096751.1**	NMU	0.229	0.010874
**AC096751.1**	RPIA	-0.217	0.011229
**AC096751.1**	RNF19B	0.228	0.011234
**AC096751.1**	MCUB	0.228	0.011234
**AC096751.1**	RNF19A	0.228	0.011234
**AC096751.1**	AK4	0.228	0.011234
**AC096751.1**	RABEP2	-0.216	0.011599
**AC096751.1**	C19orf73	-0.216	0.011599
**AC096751.1**	TMEM140	0.227	0.011605
**AC096751.1**	SMCO2	0.227	0.011605
**AC096751.1**	TEP1	0.227	0.011605
**AC096751.1**	LCLAT1	0.227	0.011605
**AC096751.1**	LEFTY1	-0.215	0.011981
**AC096751.1**	KCNN4	-0.215	0.011981
**AC096751.1**	C14orf93	-0.215	0.011981
**AC096751.1**	INPP5D	-0.215	0.011981
**AC096751.1**	FAM171A1	-0.215	0.011981
**AC096751.1**	RABEPK	-0.215	0.011981
**AC096751.1**	PERM1	0.226	0.011987
**AC096751.1**	ACHE	0.226	0.011987
**AC096751.1**	LPIN2	0.226	0.011987
**AC096751.1**	RARRES1	0.226	0.011987
**AC096751.1**	ANKH	-0.214	0.012373
**AC096751.1**	FGFR4	-0.214	0.012373
**AC096751.1**	ASPHD2	0.225	0.01238
**AC096751.1**	PLEKHA7	0.225	0.01238

**AC096751.1**	MYRFL	0.225	0.01238
**AC096751.1**	RAPH1	0.225	0.01238
**AC096751.1**	ANTXR2	0.225	0.01238
**AC096751.1**	PARP9	0.225	0.01238
**AC096751.1**	LANCL3	0.225	0.01238
**AC096751.1**	GNAI3	0.225	0.01238
**AC096751.1**	CTSE	0.225	0.01238
**AC096751.1**	AC008687.4	0.225	0.01238
**AC096751.1**	CNNM4	0.225	0.01238
**AC096751.1**	EDEM1	0.225	0.01238
**AC096751.1**	SERPINB8	0.225	0.01238
**AC096751.1**	GJA3	0.225	0.01238
**AC096751.1**	ABCA3	0.224	0.012784
**AC096751.1**	FAM114A1	0.224	0.012784
**AC096751.1**	PLCL2	0.224	0.012784
**AC096751.1**	SLMAP	0.223	0.013199
**AC096751.1**	TRIM36	0.223	0.013199
**AC096751.1**	LIPH	0.223	0.013199
**AC096751.1**	ANKRD13C	0.223	0.013199
**AC096751.1**	PATZ1	-0.211	0.01362
**AC096751.1**	CREB3L4	-0.211	0.01362
**AC096751.1**	BCL9	-0.211	0.01362
**AC096751.1**	C11orf49	-0.211	0.01362
**AC096751.1**	SNX9	0.222	0.013627
**AC096751.1**	BARX2	0.222	0.013627
**AC096751.1**	C6orf106	0.222	0.013627
**AC096751.1**	CALB1	0.222	0.013627
**AC096751.1**	TMEM63A	-0.21	0.01406
**AC096751.1**	FLAD1	-0.21	0.01406
**AC096751.1**	RPL7A	-0.21	0.01406
**AC096751.1**	BICDL1	0.221	0.014067
**AC096751.1**	HLA-C	0.221	0.014067
**AC096751.1**	ACBD6	-0.209	0.014512
**AC096751.1**	NCK2	-0.209	0.014512
**AC096751.1**	C20orf196	-0.209	0.014512
**AC096751.1**	FAM221B	-0.209	0.014512
**AC096751.1**	NDRG2	-0.209	0.014512
**AC096751.1**	KIF12	-0.209	0.014512
**AC096751.1**	MMP28	0.22	0.014519
**AC096751.1**	MTF1	0.22	0.014519
**AC096751.1**	PSEN1	0.22	0.014519
**AC096751.1**	PLK2	0.22	0.014519
**AC096751.1**	FAM102B	0.22	0.014519
**AC096751.1**	ZNF444	-0.208	0.014977

**AC096751.1**	FOXA3	-0.208	0.014977
**AC096751.1**	ACTL10	-0.208	0.014977
**AC096751.1**	GULP1	0.219	0.014984
**AC096751.1**	ERRFI1	0.219	0.014984
**AC096751.1**	ENO2	0.219	0.014984
**AC096751.1**	CCNG2	0.219	0.014984
**AC096751.1**	RUBCN	0.219	0.014984
**AC096751.1**	MT1E	0.219	0.014984
**AC096751.1**	EEPD1	-0.207	0.015455
**AC096751.1**	TMEM216	-0.207	0.015455
**AC096751.1**	PGAP2	-0.207	0.015455
**AC096751.1**	UGT1A1	0.218	0.015462
**AC096751.1**	RAP2B	0.218	0.015462
**AC096751.1**	RALBP1	0.218	0.015462
**AC096751.1**	ARNTL2	0.218	0.015462
**AC096751.1**	CYP2C18	0.218	0.015462
**AC096751.1**	TNFRSF21	0.218	0.015462
**AC096751.1**	ABCG2	0.218	0.015462
**AC096751.1**	DUSP18	-0.206	0.015946
**AC096751.1**	GFOD2	-0.206	0.015946
**AC096751.1**	ADM	0.217	0.015954
**AC096751.1**	SLC7A7	0.217	0.015954
**AC096751.1**	MICB	0.217	0.015954
**AC096751.1**	ZMYND15	0.217	0.015954
**AC096751.1**	AHCYL2	0.217	0.015954
**AC096751.1**	ALCAM	0.217	0.015954
**AC096751.1**	IRF2BP2	-0.205	0.016451
**AC096751.1**	LDB1	-0.205	0.016451
**AC096751.1**	SGMS2	0.216	0.016459
**AC096751.1**	ZNF165	0.216	0.016459
**AC096751.1**	RHOF	0.216	0.016459
**AC096751.1**	TRANK1	0.216	0.016459
**AC096751.1**	LRRFIP1	0.216	0.016459
**AC096751.1**	STARD10	-0.204	0.01697
**AC096751.1**	SLC5A6	-0.204	0.01697
**AC096751.1**	PIK3CB	0.215	0.016978
**AC096751.1**	GLI2	0.215	0.016978
**AC096751.1**	SYNJ1	0.215	0.016978
**AC096751.1**	EHBP1L1	0.215	0.016978
**AC096751.1**	CRYZ	0.215	0.016978
**AC096751.1**	TSPAN1	0.215	0.016978
**AC096751.1**	SSFA2	0.215	0.016978
**AC096751.1**	RCAN1	0.215	0.016978
**AC096751.1**	MNX1	-0.203	0.017503

**AC096751.1**	MEX3A	-0.203	0.017503
**AC096751.1**	SLC25A26	-0.203	0.017503
**AC096751.1**	ANKLE2	0.214	0.017511
**AC096751.1**	ITPRIPL2	0.214	0.017511
**AC096751.1**	CRYBA4	0.214	0.017511
**AC096751.1**	ETV7	0.214	0.017511
**AC096751.1**	GAREM1	0.214	0.017511
**AC096751.1**	SGMS1	0.214	0.017511
**AC096751.1**	AHR	0.214	0.017511
**AC096751.1**	BTNL8	0.214	0.017511
**AC096751.1**	PLEKHN1	0.214	0.017511
**AC096751.1**	SLC2A8	-0.202	0.018051
**AC096751.1**	MAP1LC3A	-0.202	0.018051
**AC096751.1**	TRIM68	-0.202	0.018051
**AC096751.1**	ZSCAN2	-0.202	0.018051
**AC096751.1**	SEPHS1	-0.202	0.018051
**AC096751.1**	RAET1E	0.213	0.01806
**AC096751.1**	RALB	0.213	0.01806
**AC096751.1**	AKR1B10	0.213	0.01806
**AC096751.1**	COL4A3BP	0.213	0.01806
**AC096751.1**	PRDX5	-0.201	0.018614
**AC096751.1**	MSRB2	-0.201	0.018614
**AC096751.1**	CHRM3	-0.201	0.018614
**AC096751.1**	SPAG9	0.212	0.018623
**AC096751.1**	NAPG	0.212	0.018623
**AC096751.1**	FSTL3	0.212	0.018623
**AC096751.1**	ROCK1	0.212	0.018623
**AC096751.1**	RPL15	-0.2	0.019192
**AC096751.1**	CAT	-0.2	0.019192
**AC096751.1**	SMTN	-0.2	0.019192
**AC096751.1**	ARHGEF19	-0.2	0.019192
**AC096751.1**	POLR3C	-0.2	0.019192
**AC096751.1**	NCOA2	0.211	0.019201
**AC096751.1**	CDCP1	0.211	0.019201
**AC096751.1**	IL6R	0.211	0.019201
**AC096751.1**	MAP3K6	0.211	0.019201
**AC096751.1**	BDKRB1	0.211	0.019201
**AC096751.1**	CDC42BPA	0.211	0.019201
**AC096751.1**	MAX	0.211	0.019201
**AC096751.1**	CAB39	0.211	0.019201
**AC096751.1**	ACTBL2	0.211	0.019201
**AC096751.1**	HLA-E	0.211	0.019201
**AC096751.1**	MUC17	0.211	0.019201
**AC096751.1**	HIPK1	0.21	0.019795

**AC096751.1**	DENND1C	0.21	0.019795
**AC096751.1**	SMPDL3A	0.21	0.019795
**AC096751.1**	C11orf86	0.21	0.019795
**AC096751.1**	BMP8B	0.21	0.019795
**AC096751.1**	CHMP2B	0.21	0.019795
**AC096751.1**	SEC24A	0.21	0.019795
**AC096751.1**	RNF144A	0.21	0.019795
**AC096751.1**	CAV1	0.209	0.020405
**AC096751.1**	SECTM1	0.209	0.020405
**AC096751.1**	UBR2	0.209	0.020405
**AC096751.1**	DSC2	0.209	0.020405
**AC096751.1**	SPATA5	0.209	0.020405
**AC096751.1**	PLEC	0.209	0.020405
**AC096751.1**	MAFF	0.209	0.020405
**AC096751.1**	AHNAK2	0.209	0.020405
**AC096751.1**	BCL10	0.208	0.021031
**AC096751.1**	TMBIM1	0.208	0.021031
**AC096751.1**	RAB12	0.208	0.021031
**AC096751.1**	RPS6KA5	0.208	0.021031
**AC096751.1**	ITIH4	0.208	0.021031
**AC096751.1**	SPATS2L	0.208	0.021031
**AC096751.1**	FEZ2	0.208	0.021031
**AC096751.1**	EDN3	0.208	0.021031
**AC096751.1**	ZCCHC2	0.208	0.021031
**AC096751.1**	HRASLS2	0.208	0.021031
**AC096751.1**	SI	0.208	0.021031
**AC096751.1**	IRF8	0.208	0.021031
**AC096751.1**	TFAP2A	0.207	0.021674
**AC096751.1**	PDE4D	0.207	0.021674
**AC096751.1**	PTPRU	0.207	0.021674
**AC096751.1**	PLLP	0.207	0.021674
**AC096751.1**	SLC2A5	0.207	0.021674
**AC096751.1**	S100A16	0.207	0.021674
**AC096751.1**	SGPP2	0.207	0.021674
**AC096751.1**	SP110	0.206	0.022335
**AC096751.1**	EPS8	0.206	0.022335
**AC096751.1**	IGSF5	0.206	0.022335
**AC096751.1**	CYTH1	0.206	0.022335
**AC096751.1**	S100A10	0.206	0.022335
**AC096751.1**	RBBP8	0.206	0.022335
**AC096751.1**	JAK2	0.205	0.023012
**AC096751.1**	ZNFX1	0.205	0.023012
**AC096751.1**	EVI5	0.205	0.023012
**AC096751.1**	IFIT5	0.205	0.023012

**AC096751.1**	LMO7	0.205	0.023012
**AC096751.1**	ATP2B1	0.205	0.023012
**AC096751.1**	BBS7	0.205	0.023012
**AC096751.1**	MTUS1	0.204	0.023707
**AC096751.1**	TBC1D1	0.204	0.023707
**AC096751.1**	RBMXL1	0.204	0.023707
**AC096751.1**	ME1	0.204	0.023707
**AC096751.1**	NR3C2	0.204	0.023707
**AC096751.1**	BMP2	0.204	0.023707
**AC096751.1**	MXI1	0.203	0.024421
**AC096751.1**	SLC9A7	0.203	0.024421
**AC096751.1**	AARD	0.203	0.024421
**AC096751.1**	ASS1	0.203	0.024421
**AC096751.1**	CDA	0.203	0.024421
**AC096751.1**	RPE65	0.203	0.024421
**AC096751.1**	STAT1	0.203	0.024421
**AC096751.1**	SPATA46	0.203	0.024421
**AC096751.1**	FP565260.3	0.203	0.024421
**AC096751.1**	LCOR	0.202	0.025153
**AC096751.1**	ZDHHC18	0.202	0.025153
**AC096751.1**	ARHGEF18	0.202	0.025153
**AC096751.1**	LAMA3	0.202	0.025153
**AC096751.1**	RAMP1	0.202	0.025153
**AC096751.1**	TPCN2	0.202	0.025153
**AC096751.1**	DTX3L	0.202	0.025153
**AC096751.1**	AC020914.1	0.2	0.026674
**AC096751.1**	NKX3-1	0.2	0.026674
**AC096751.1**	KCNIP1	0.2	0.026674
**AC096751.1**	HHLA2	0.2	0.026674
**AC096751.1**	SLC45A4	0.2	0.026674
**AC096751.1**	CLCF1	0.2	0.026674
**AC096751.1**	PDP1	0.2	0.026674
**AC096751.1**	HLA-B	0.2	0.026674
**AC096751.1**	NCR1	0.2	0.026674
**AC096751.1**	FECH	0.2	0.026674

Table S3.Survival analysis results of AC096751.1 co-expressed genes (Only gene list with P<0.05 is shown).

ID	P	HR	Low 95%CI	High 95%CI
**DAPK1**	0.002093399	1.946256078	1.273405483	2.974632018
**EVI5**	0.00210591	0.502695535	0.324274713	0.779286176
**CRYBA4**	0.004681786	1.848958507	1.20765074	2.830824713
**KCTD1**	0.00826329	1.766290773	1.158025855	2.694053055
**DPCR1**	0.010646043	1.74395656	1.138116395	2.67229652
**ENO2**	0.011888209	1.729654848	1.128621112	2.650761943
**PHLPP2**	0.018535165	0.605717061	0.39906461	0.919382848
**SLMAP**	0.019496164	0.610854644	0.403949433	0.923737888
**BCL10**	0.020595917	0.607591107	0.398504742	0.926380327
**CYP39A1**	0.021761359	0.61571128	0.406877283	0.931731499
**RAPH1**	0.02180111	0.611877998	0.402141925	0.931001374
**RPS6KA5**	0.022551623	0.610973258	0.400088503	0.93301437
**HIPK1**	0.024819195	0.622342649	0.411288391	0.94170023
**FGFR4**	0.026558587	0.619632194	0.405925141	0.945849413
**GNA13**	0.027605398	0.626467405	0.413229031	0.949743073
**NMU**	0.031748336	1.585489847	1.041082907	2.414580088
**TGFBI**	0.035851527	0.639686146	0.42145633	0.970915221
**SLC2A5**	0.040183693	1.542052002	1.019586205	2.332244556
**ADAM9**	0.04050391	0.647144756	0.426754196	0.98135259
**SEC24A**	0.041295492	0.648328635	0.427585651	0.98303116
**EFNA4**	0.042195168	0.650934846	0.430178796	0.984976894
**CHMP2B**	0.043383665	0.653217658	0.432127815	0.987423846
**SLC25A26**	0.045173679	0.656052247	0.434323254	0.990977449

Table S4. Functional enrichment analysis results of AC096751.1 co-expressed genes

Category	Term	Count	P Value
**KEGG_PATHWAY**	hsa05200:Pathways in cancer	24	4.10E-04
**KEGG_PATHWAY**	hsa05168:Herpes simplex infection	15	4.55E-04
**KEGG_PATHWAY**	hsa05222:Small cell lung cancer	9	0.002137336
**KEGG_PATHWAY**	hsa05416:Viral myocarditis	7	0.004386428
**KEGG_PATHWAY**	hsa04668:TNF signaling pathway	9	0.008713227
**KEGG_PATHWAY**	hsa05332:Graft-versus-host disease	5	0.011899509
**KEGG_PATHWAY**	hsa04612:Antigen processing and presentation	7	0.017281288
**KEGG_PATHWAY**	hsa05330:Allograft rejection	5	0.017653712
**KEGG_PATHWAY**	hsa04940:Type I diabetes mellitus	5	0.026940128
**KEGG_PATHWAY**	hsa04810:Regulation of actin cytoskeleton	12	0.029018217
**KEGG_PATHWAY**	hsa05145:Toxoplasmosis	8	0.030561909
**KEGG_PATHWAY**	hsa02010:ABC transporters	5	0.031336006
**KEGG_PATHWAY**	hsa00600:Sphingolipid metabolism	5	0.038676926
**KEGG_PATHWAY**	hsa05140:Leishmaniasis	6	0.044129353
**KEGG_PATHWAY**	hsa04071:Sphingolipid signaling pathway	8	0.045659678
**GOTERM_CC_DIRECT**	GO:0045121~membrane raft	24	1.37E-09
**GOTERM_BP_DIRECT**	GO:0060333~interferon-gamma-mediated signaling pathway	13	1.60E-07
**GOTERM_BP_DIRECT**	GO:0007165~signal transduction	58	5.81E-07
**GOTERM_CC_DIRECT**	GO:0005886~plasma membrane	144	1.90E-06
**GOTERM_CC_DIRECT**	GO:0070062~extracellular exosome	102	2.79E-05
**GOTERM_CC_DIRECT**	GO:0005765~lysosomal membrane	20	4.70E-05
**GOTERM_CC_DIRECT**	GO:0048471~perinuclear region of cytoplasm	33	6.16E-05
**GOTERM_MF_DIRECT**	GO:0005102~receptor binding	23	7.63E-05
**GOTERM_CC_DIRECT**	GO:0005737~cytoplasm	165	9.32E-05
**GOTERM_MF_DIRECT**	GO:0005515~protein binding	257	9.73E-05
**GOTERM_CC_DIRECT**	GO:0016324~apical plasma membrane	20	1.05E-04
**GOTERM_CC_DIRECT**	GO:0000139~Golgi membrane	30	3.14E-04
**GOTERM_CC_DIRECT**	GO:0005856~cytoskeleton	22	3.33E-04
**GOTERM_CC_DIRECT**	GO:0005794~Golgi apparatus	39	3.35E-04
**GOTERM_MF_DIRECT**	GO:0042803~protein homodimerization activity	35	3.38E-04
**GOTERM_CC_DIRECT**	GO:0005913~cell-cell adherens junction	20	3.94E-04
**GOTERM_BP_DIRECT**	GO:0043547~positive regulation of GTPase activity	29	4.32E-04
**GOTERM_BP_DIRECT**	GO:0002474~antigen processing and presentation of peptide antigen via MHC class I	6	7.87E-04
**GOTERM_BP_DIRECT**	GO:0006939~smooth muscle contraction	5	8.70E-04
**GOTERM_BP_DIRECT**	GO:0060337~type I interferon signaling pathway	8	0.001016401
**GOTERM_BP_DIRECT**	GO:0032436~positive regulation of proteasomal ubiquitin-dependent protein catabolic process	8	0.001016401
**GOTERM_CC_DIRECT**	GO:0005829~cytosol	108	0.001034324
**GOTERM_CC_DIRECT**	GO:0016020~membrane	77	0.001075629
**GOTERM_BP_DIRECT**	GO:0030148~sphingolipid biosynthetic process	7	0.001148912
**GOTERM_BP_DIRECT**	GO:0002250~adaptive immune response	12	0.00118282

**GOTERM_CC_DIRECT**	GO:0005768~endosome	15	0.001319108
**GOTERM_CC_DIRECT**	GO:0005764~lysosome	15	0.001375498
**GOTERM_MF_DIRECT**	GO:0008289~lipid binding	12	0.001386535
**GOTERM_MF_DIRECT**	GO:0005125~cytokine activity	13	0.001512396
**GOTERM_CC_DIRECT**	GO:0005925~focal adhesion	21	0.001584375
**GOTERM_BP_DIRECT**	GO:0050714~positive regulation of protein secretion	6	0.001842935
**GOTERM_BP_DIRECT**	GO:0006915~apoptotic process	27	0.002059684
**GOTERM_BP_DIRECT**	GO:0008283~cell proliferation	20	0.002097451
**GOTERM_BP_DIRECT**	GO:0035970~peptidyl-threonine dephosphorylation	4	0.00216249
**GOTERM_MF_DIRECT**	GO:0098641~cadherin binding involved in cell-cell adhesion	17	0.002509195
**GOTERM_BP_DIRECT**	GO:0007173~epidermal growth factor receptor signaling pathway	7	0.002574223
**GOTERM_BP_DIRECT**	GO:0070373~negative regulation of ERK1 and ERK2 cascade	7	0.003078721
**GOTERM_BP_DIRECT**	GO:0034612~response to tumor necrosis factor	5	0.003135578
**GOTERM_CC_DIRECT**	GO:0005903~brush border	7	0.003369631
**GOTERM_BP_DIRECT**	GO:0031018~endocrine pancreas development	5	0.003634017
**GOTERM_BP_DIRECT**	GO:0016477~cell migration	12	0.003853907
**GOTERM_MF_DIRECT**	GO:0042605~peptide antigen binding	5	0.004778395
**GOTERM_CC_DIRECT**	GO:0016323~basolateral plasma membrane	12	0.004803495
**GOTERM_MF_DIRECT**	GO:0004871~signal transducer activity	13	0.005062333
**GOTERM_BP_DIRECT**	GO:0071549~cellular response to dexamethasone stimulus	5	0.005445393
**GOTERM_BP_DIRECT**	GO:0000186~activation of MAPKK activity	6	0.005473974
**GOTERM_BP_DIRECT**	GO:0009267~cellular response to starvation	6	0.006003441
**GOTERM_CC_DIRECT**	GO:0031234~extrinsic component of cytoplasmic side of plasma membrane	7	0.006261178
**GOTERM_BP_DIRECT**	GO:0006810~transport	18	0.006351654
**GOTERM_CC_DIRECT**	GO:0009986~cell surface	24	0.007516947
**GOTERM_BP_DIRECT**	GO:0008285~negative regulation of cell proliferation	19	0.010241769
**GOTERM_CC_DIRECT**	GO:0046581~intercellular canaliculus	3	0.011517577
**GOTERM_BP_DIRECT**	GO:0019882~antigen processing and presentation	6	0.011593807
**GOTERM_BP_DIRECT**	GO:0050852~T cell receptor signaling pathway	10	0.011691908
**GOTERM_MF_DIRECT**	GO:0008134~transcription factor binding	15	0.011747008
**GOTERM_CC_DIRECT**	GO:0016021~integral component of membrane	149	0.012082895
**GOTERM_MF_DIRECT**	GO:0046983~protein dimerization activity	10	0.012647885
**GOTERM_CC_DIRECT**	GO:0030176~integral component of endoplasmic reticulum membrane	8	0.01380885
**GOTERM_BP_DIRECT**	GO:0042517~positive regulation of tyrosine phosphorylation of Stat3 protein	5	0.014213954
**GOTERM_BP_DIRECT**	GO:0071407~cellular response to organic cyclic compound	6	0.015402059
**GOTERM_BP_DIRECT**	GO:0006953~acute-phase response	5	0.015535716
**GOTERM_MF_DIRECT**	GO:0005518~collagen binding	6	0.016438932
**GOTERM_BP_DIRECT**	GO:0048661~positive regulation of smooth muscle cell proliferation	6	0.016470103
**GOTERM_BP_DIRECT**	GO:0010508~positive regulation of autophagy	5	0.016932325

**GOTERM_BP_DIRECT**	GO:0098609~cell-cell adhesion	14	0.018244476
**GOTERM_MF_DIRECT**	GO:0046934~phosphatidylinositol-4,5-bisphosphate 3-kinase activity	6	0.01871617
**GOTERM_MF_DIRECT**	GO:0046703~natural killer cell lectin-like receptor binding	3	0.019712865
**GOTERM_BP_DIRECT**	GO:0002480~antigen processing and presentation of exogenous peptide antigen via MHC class I, TAP-independent	3	0.019731328
**GOTERM_CC_DIRECT**	GO:0005615~extracellular space	46	0.01976956
**GOTERM_BP_DIRECT**	GO:0006979~response to oxidative stress	8	0.019828792
**GOTERM_CC_DIRECT**	GO:0005901~caveola	6	0.021162436
**GOTERM_BP_DIRECT**	GO:0030155~regulation of cell adhesion	5	0.021582427
**GOTERM_BP_DIRECT**	GO:0031623~receptor internalization	5	0.021582427
**GOTERM_MF_DIRECT**	GO:0042626~ATPase activity, coupled to transmembrane movement of substances	5	0.023252335
**GOTERM_MF_DIRECT**	GO:0043236~laminin binding	4	0.023292493
**GOTERM_BP_DIRECT**	GO:0015949~nucleobase-containing small molecule interconversion	4	0.023322355
**GOTERM_BP_DIRECT**	GO:0000188~inactivation of MAPK activity	4	0.023322355
**GOTERM_MF_DIRECT**	GO:0019911~structural constituent of myelin sheath	3	0.024241121
**GOTERM_BP_DIRECT**	GO:0008015~blood circulation	5	0.025075547
**GOTERM_BP_DIRECT**	GO:0007267~cell-cell signaling	13	0.025265057
**GOTERM_BP_DIRECT**	GO:0032355~response to estradiol	7	0.025748741
**GOTERM_BP_DIRECT**	GO:0042552~myelination	5	0.026942364
**GOTERM_CC_DIRECT**	GO:0042612~MHC class I protein complex	3	0.028281188
**GOTERM_CC_DIRECT**	GO:0043296~apical junction complex	3	0.028281188
**GOTERM_BP_DIRECT**	GO:0071230~cellular response to amino acid stimulus	5	0.02889017
**GOTERM_BP_DIRECT**	GO:0070166~enamel mineralization	3	0.029175003
**GOTERM_BP_DIRECT**	GO:0007175~negative regulation of epidermal growth factor-activated receptor activity	3	0.029175003
**GOTERM_BP_DIRECT**	GO:0046854~phosphatidylinositol phosphorylation	7	0.029592511
**GOTERM_CC_DIRECT**	GO:0005813~centrosome	18	0.032967339
**GOTERM_BP_DIRECT**	GO:0036092~phosphatidylinositol-3-phosphate biosynthetic process	5	0.033030657
**GOTERM_CC_DIRECT**	GO:0071556~integral component of lumenal side of endoplasmic reticulum membrane	4	0.033033828
**GOTERM_CC_DIRECT**	GO:0005789~endoplasmic reticulum membrane	31	0.033407473
**GOTERM_CC_DIRECT**	GO:0043231~intracellular membrane-bounded organelle	22	0.033656529
**GOTERM_BP_DIRECT**	GO:0006955~immune response	18	0.034076904
**GOTERM_MF_DIRECT**	GO:0000983~transcription factor activity, RNA polymerase II core promoter sequence-specific	3	0.034412471
**GOTERM_BP_DIRECT**	GO:0030212~hyaluronan metabolic process	3	0.0344439
**GOTERM_BP_DIRECT**	GO:0071985~multivesicular body sorting pathway	3	0.0344439
**GOTERM_BP_DIRECT**	GO:0031581~hemidesmosome assembly	3	0.0344439
**GOTERM_MF_DIRECT**	GO:0019904~protein domain specific binding	11	0.034887836
**GOTERM_CC_DIRECT**	GO:0030659~cytoplasmic vesicle membrane	8	0.035039436

**GOTERM_BP_DIRECT**	GO:0006468~protein phosphorylation	19	0.036113396
**GOTERM_CC_DIRECT**	GO:0031982~vesicle	8	0.037669909
**GOTERM_CC_DIRECT**	GO:0012507~ER to Golgi transport vesicle membrane	5	0.03785539
**GOTERM_BP_DIRECT**	GO:0034097~response to cytokine	5	0.039858342
**GOTERM_BP_DIRECT**	GO:0016337~single organismal cell-cell adhesion	7	0.039972213
**GOTERM_MF_DIRECT**	GO:0017017~MAP kinase tyrosine/serine/threonine phosphatase activity	3	0.040013397
**GOTERM_BP_DIRECT**	GO:0048546~digestive tract morphogenesis	3	0.040049631
**GOTERM_BP_DIRECT**	GO:0010288~response to lead ion	3	0.040049631
**GOTERM_BP_DIRECT**	GO:0042981~regulation of apoptotic process	11	0.04040461
**GOTERM_BP_DIRECT**	GO:0030324~lung development	6	0.040561252
**GOTERM_BP_DIRECT**	GO:0006869~lipid transport	6	0.040561252
**GOTERM_BP_DIRECT**	GO:0000209~protein polyubiquitination	10	0.04056262
**GOTERM_CC_DIRECT**	GO:0005887~integral component of plasma membrane	46	0.04136926
**GOTERM_BP_DIRECT**	GO:0007259~JAK-STAT cascade	4	0.044320235
**GOTERM_BP_DIRECT**	GO:0007498~mesoderm development	4	0.044320235
**GOTERM_BP_DIRECT**	GO:0014066~regulation of phosphatidylinositol 3-kinase signaling	6	0.044558434
**GOTERM_MF_DIRECT**	GO:0019003~GDP binding	5	0.04475615
**GOTERM_BP_DIRECT**	GO:0007411~axon guidance	9	0.045125926
**GOTERM_BP_DIRECT**	GO:0003407~neural retina development	3	0.045972305
**GOTERM_BP_DIRECT**	GO:0071364~cellular response to epidermal growth factor stimulus	4	0.047884246
**GOTERM_BP_DIRECT**	GO:0043123~positive regulation of I-kappaB kinase/NF-kappaB signaling	9	0.047945551
**GOTERM_BP_DIRECT**	GO:0048015~phosphatidylinositol-mediated signaling	7	0.048641276
**GOTERM_BP_DIRECT**	GO:0046967~cytosol to ER transport	2	0.04905126
**GOTERM_CC_DIRECT**	GO:0001772~immunological synapse	4	0.049514809

Table S5.GSEA analysis results between high - and low -AC096751.1 phenotypes in COAD

**NAME**	**SIZE**	**ES**	**NES**	**NOM p-val**	**FDR q-val**
**GOBP_MITOCHONDRIAL_ELECTRON_TRANSPORT_CYTOCHROME_C_TO_OXYGEN**	19	0.80975765	1.8951869	<0.01	0.24090937
**GOBP_PYRIMIDINE_RIBONUCLEOSIDE_TRIPHOSPHATE_METABOLIC_PROCESS**	18	0.65890646	1.8834474	0.001996008	0.20947339
**GOBP_RIBOSOMAL_SMALL_SUBUNIT_ASSEMBLY**	19	0.795074	1.8794417	0.002008032	0.2010365
**HP_MACROCYTIC_ANEMIA**	56	0.60406446	1.9071213	0.002008032	0.24952225
**HP_ABNORMALITY_OF_THE_MUSCULATURE_OF_THE_HAND**	86	0.48520508	1.884091	0.00204499	0.2210944
**GOBP_PYRIMIDINE_NUCLEOTIDE_METABOLIC_PROCESS**	49	0.5522471	1.8774265	0.003853565	0.19496028
**GOCC_SMALL_RIBOSOMAL_SUBUNIT**	71	0.74180776	1.8859416	0.003984064	0.23934138
**HP_ERYTHROID_HYPOPLASIA**	24	0.8169176	1.8959482	0.005758158	0.24860276
**HP_PERSISTENCE_OF_HEMOGLOBIN_F**	28	0.7473389	1.869834	0.005825243	0.19034462
**HP_DEVELOPMENTAL_GLAUCOMA**	60	0.5488402	1.8760072	0.00591716	0.18804759
**HP_PARTIAL_DUPLICATION_OF_THE_PHALANX_OF_HAND**	38	0.62397784	1.902742	0.007736944	0.240628
**HP_ELEVATED_RED_CELL_ADENOSINE_DEAMINASE_LEVEL**	21	0.8364585	1.8197936	0.007751938	0.22083934
**HP_NONIMMUNE_HYDROPS_FETALIS**	40	0.668355	1.8779552	0.007843138	0.19891496

**HP_SPRENGEL_ANOMALY**	49	0.5791	1.8853	0.00796	0.2326
		8614	256	8128	9795
**GOCC_RESPIRASOME**	97	0.7015	1.9069	0.00796	0.2390
		9495	755	8128	6949
**GOCC_LARGE_RIBOSOMAL_SUBUNIT**	114	0.7398	1.8796	0.00798	0.2062
		391	082	4032	7543
**GOBP_GLYCOSYL_COMPOUND_BIOSYNTHETIC_PROCESS**	40	0.6155	1.8843	0.008	0.2273
		5165	865		2924
**GOBP_POSITIVE_REGULATION_OF_VIRAL_TRANSCRIPTION**	26	0.6461	1.8925	0.01008	0.2384
		892	848	0645	8051
**HP_MALIGNANT_GENITOURINARY_TRACT_TUMOR**	25	0.7796	1.8254	0.01158	0.2129
		479	523	3012	4136
**HP_PURE_RED_CELL_APLASIA**	24	0.7858	1.8163	0.01158	0.2208
		8164	51	3012	4205
**GOBP_PYRIMIDINE_NUCLEOTIDE_BIOSYNTHETIC_PROCESS**	29	0.6148	1.8372	0.01169	0.2107
		1655	493	5907	2827
**GOCC_CYTOCHROME_COMPLEX**	35	0.7108	1.8537	0.012	0.2052
		879	82		9208
**HP_APLASIA_OF_THE_FINGERS**	65	0.5068	1.8170	0.012	0.2230
		1686	01		8485
**HP_THROMBOCYTOSIS**	48	0.5769	1.8474	0.01207	0.2103
		792	312	2435	4196
**HP_INCREASED_MEAN_CORPUSCULAR_VOLUME**	36	0.6408	1.8373	0.01377	0.2145
		331	37	9528	4826
**GOCC_RESPIRATORY_CHAIN_COMPLEX**	83	0.7057	1.8836	0.01380	0.2153
		394	89	6706	4465
**GOBP_PYRIMIDINE_RIBONUCLEOTIDE_BIOSYNTHETIC_PROCESS**	22	0.6492	1.8457	0.01386	0.2097
		74	6	1386	2957
**GOBP_ATP_SYNTHESIS_COUPLED_ELECTRON_TRANSPORT**	97	0.7013	1.8657	0.01397	0.1899

		052	482	2056	5367
**GOBP_TRANSLATIONAL_INITIATION**	187	0.6253	1.8907	0.01541	0.2346
		4344	079	4258	656
**HP_DUPLICATION_OF_THUMB_PHALANX**	52	0.5609	1.8402	0.01596	0.2127
		048	189	8064	6551
**HP_RETICULOCYTOPENIA**	33	0.6592	1.8338	0.01714	0.2100
		152	314	2856	4379
**HP_ABSENT_THUMB**	43	0.5928	1.8061	0.01778	0.2245
		2184	135	656	7473
**GOBP_NUCLEAR_TRANSCRIBED_MRNA_CATABOLIC_PROCESS_NONSENSE_MEDIATED_DECA**	120	0.6876	1.8699	0.01785	0.1946
**Y**		306	847	7144	0963
**GOCC_NADH_DEHYDROGENASE_COMPLEX**	47	0.7240	1.8095	0.01785	0.2242
		4844	207	7144	4042
**GOMF_HEME_COPPER_TERMINAL_OXIDASE_ACTIVITY**	27	0.7138	1.8410	0.01792	0.2149
		9174	796	8287	5976
**GOCC_POLYSOME**	68	0.5861	1.8118	0.01953	0.2229
		052	67	125	9328
**GOBP_RIBOSOME_ASSEMBLY**	59	0.6545	1.8148	0.01980	0.2205
		0543	459	198	792
**HP_OSTEOSARCOMA**	32	0.6886	1.8510	0.02067	0.2067
		585	729	6691	493
**GOBP_PROTEIN_LOCALIZATION_TO_ENDOPLASMIC_RETICULUM**	142	0.6407	1.8588	0.02148	0.1994
		394	123	4375	3988
**HP_ABNORMAL_NUMBER_OF_ERYTHROID_PRECURSORS**	35	0.6831	1.8329	0.02169	0.2012
		0463	296	6253	5581
**GOBP_NUCLEAR_TRANSCRIBED_MRNA_CATABOLIC_PROCESS**	199	0.5579	1.8339	0.02325	0.2136
		004	305	5814	806
**GOCC_INNER_MITOCHONDRIAL_MEMBRANE_PROTEIN_COMPLEX**	115	0.6654	1.8804	0.02366	0.2101
		8157	523	864	2625

**GOMF_RRNA_BINDING**	59	0.6754	1.8336	0.024	0.2066
		749	972		01
**KIM_ALL_DISORDERS_DURATION_CORR_DN**	140	0.6540	1.9901	0.00200	0.1498
		722	317	4008	4359
**REACTOME_CELLULAR_RESPONSE_TO_STARVATION**	152	0.7049	2.0064	0.00610	0.1528
		533	285	998	479
**POMEROY_MEDULLOBLASTOMA_PROGNOSIS_DN**	41	0.7308	2.0160	0.00396	0.1538
		307	6	0396	171
**ABE_INNER_EAR**	40	0.6629	1.9954	<0.01	0.1565
		771	26		3266
**BILANGES_SERUM_RESPONSE_TRANSLATION**	31	0.7599	2.0213	<0.01	0.1670
		049	73		9052
**PECE_MAMMARY_STEM_CELL_UP**	129	0.6893	2.0873	<0.01	0.1765
		1526	373		1685
**REACTOME_SELENOAMINO_ACID_METABOLISM**	108	0.7819	1.9385	0.00606	0.1793
		981	908	0606	4799
**REACTOME_SIGNALING_BY_ROBO_RECEPTORS**	211	0.5846	1.9180	0.01375	0.1814
		635	821	2456	2438
**REACTOME_METABOLISM_OF_AMINO_ACIDS_AND_DERIVATIVES**	351	0.5553	1.9417	0.01192	0.1843
		761	307	843	0115
**REACTOME_ABORTIVE_ELONGATION_OF_HIV_1_TRANSCRIPT_IN_THE_ABSENCE_OF_TAT**	23	0.7153	1.9203	0.00787	0.1862
		411	76	4016	203
**REACTOME_SULFUR_AMINO_ACID_METABOLISM**	27	0.6797	2.0255	<0.01	0.1888
		315	697		2619
**IRITANI_MAD1_TARGETS_DN**	44	0.6950	1.9038	0.00766	0.1929
		7444	482	2835	095
**HOLLEMAN_ASPARAGINASE_RESISTANCE_B_ALL_UP**	24	0.7625	1.9218	0.00580	0.1935
		29	668	2708	6276
**BILANGES_SERUM_AND_RAPAMYCIN_SENSITIVE_GENES**	65	0.7924	1.9427	0.00397	0.1951

		459	29	6143	6295
**LU_EZH2_TARGETS_UP**	266	0.5557	1.9068	0.00986	0.1961
		083	847	1933	1442
**LI_DCP2_BOUND_MRNA**	86	0.6638	1.9477	<0.01	0.1984
		779	97		0229
**REACTOME_RESPONSE_OF_EIF2AK4_GCN2_TO_AMINO_ACID_DEFICIENCY**	101	0.7863	1.8972	0.00801	0.1994
		9054	919	6032	1425
**REACTOME_GLYCOGEN_STORAGE_DISEASES**	16	0.6988	1.9237	<0.01	0.1996
		649	996		9879
**HOLLEMAN_VINCRISTINE_RESISTANCE_ALL_DN**	20	0.8265	2.1197	<0.01	0.2036
		086	588		4022
**REACTOME_EUKARYOTIC_TRANSLATION_INITIATION**	119	0.7648	1.8875	0.01	0.2037
		525	512		6892
**SCHLOSSER_MYC_AND_SERUM_RESPONSE_SYNERGY**	31	0.7730	1.9587	<0.01	0.2053
		823	839		3678
**MODY_HIPPOCAMPUS_PRENATAL**	40	0.7057	1.9519	0.00584	0.2055
		355	062	7953	3882
**REACTOME_NONSENSE_MEDIATED_DECAY_NMD**	115	0.7192	1.8898	0.01964	0.2068
		848	947	6365	6089
**KEGG_RNA_POLYMERASE**	27	0.7189	2.0328	0.00194	0.2110
		6124	858	1748	4845
**REACTOME_REGULATION_OF_EXPRESSION_OF_SLITS_AND_ROBOS**	167	0.6611	1.8800	0.01789	0.2116
		499	644	2644	7171
**SANSOM_APC_TARGETS_REQUIRE_MYC**	211	0.5263	1.8758	0.00588	0.2124
		152	723	2353	5995
**YAMASHITA_LIVER_CANCER_WITH_EPCAM_UP**	53	0.6332	1.8616	0.01553	0.2135
		689	396	3981	7
**CHNG_MULTIPLE_MYELOMA_HYPERPLOID_UP**	53	0.8681	2.0510	<0.01	0.2163
		068	442		1287

**REACTOME_INFLUENZA_INFECTION**	155	0.6626<br>491	1.8695<br>244	0.01832<br>9939	0.2183<br>0134
**LEE_LIVER_CANCER_MYC_UP**	50	0.5221<br>27	1.8616<br>784	<0.01	0.2206<br>3102
**KEGG_HUNTINGTONS_DISEASE**	161	0.5381<br>4065	1.8552<br>914	0.00988<br>1423	0.2206<br>4999
**WP_CYTOPLASMIC_RIBOSOMAL_PROTEINS**	88	0.8081<br>169	1.8486<br>186	0.00608<br>5193	0.2211<br>3793
**ANDERSEN_LIVER_CANCER_KRT19_UP**	34	0.7039<br>739	1.8510<br>748	0.00595<br>2381	0.2226<br>4992
**HEDENFALK_BREAST_CANCER_BRACX_UP**	18	0.6628<br>1587	1.8617<br>351	0.00617<br>284	0.2280<br>4987
**REACTOME_EUKARYOTIC_TRANSLATION_ELONGATION**	93	0.8152<br>0456	1.8366<br>524	0.00617<br>284	0.2408<br>7116
**REACTOME_ACTIVATION_OF_THE_MRNA_UPON_BINDING_OF_THE_CAP_BINDING_COMPLEX_AND_EIFS_AND_SUBSEQUENT_BINDING_TO_43S**	59	0.7443<br>236	1.8269<br>695	0.01603<br>2064	0.2419<br>3038
**KEGG_RIBOSOME**	87	0.8197<br>25	1.8240<br>659	0.00609<br>7561	0.2420<br>3016
**REACTOME_SRP_DEPENDENT_COTRANSLATIONAL_PROTEIN_TARGETING_TO_MEMBRANE**	112	0.7434<br>9856	1.8287<br>617	0.026	0.2444<br>8845
**REACTOME_RNA_POLYMERASE_III_CHAIN_ELONGATION**	18	0.6752<br>262	1.8306<br>139	0.00596<br>4215	0.2471<br>056

Table S6. DEG between high - and low -AC096751.1 phenotypes in COAD

ID	Log FC	Log CPM	P value	FDR
**PAEP**	5.455820773	3.325170379	2.14E-51	3.76E-47
**NNAT**	-2.462139553	2.406100716	4.35E-35	3.81E-31
**CHRNA4**	3.87875452	-2.832738107	5.15E-29	3.01E-25
**HPGD**	-1.380231843	5.085415306	9.06E-29	3.97E-25
**SFTPC**	-5.868055242	0.157546519	1.19E-28	4.19E-25
**UTS2B**	-2.238340142	-1.921312495	1.75E-26	5.11E-23
**CALCA**	3.163409564	2.10559192	8.99E-26	2.25E-22
**COL2A1**	2.822532384	2.055521233	3.13E-25	6.85E-22
**TFAP2B**	4.814463555	-2.05169447	5.07E-25	9.87E-22
**COLEC10**	-1.901340754	-1.217024581	1.72E-24	3.02E-21
**CLEC2A**	4.189372656	-2.663736615	7.42E-24	1.18E-20
**GCG**	-3.277575242	1.721928869	1.84E-22	2.68E-19
**NEUROD4**	3.766214905	-3.476906121	6.68E-21	9.01E-18
**ZNF479**	3.899499629	-3.281261692	1.19E-20	1.49E-17
**UGT3A1**	3.572165938	-2.997036039	1.38E-20	1.62E-17
**IGDCC3**	2.563253926	-0.993600729	7.39E-20	8.09E-17
**SULT1C4**	1.946011809	1.66166825	2.22E-19	2.29E-16
**DPCR1**	-2.474726603	-0.641051447	5.47E-19	5.33E-16
**NMRK2**	3.282380553	-2.694089472	1.01E-18	9.30E-16
**LHX3**	2.858201606	-2.490324291	1.61E-18	1.41E-15
**GALP**	3.51559122	-3.499260293	1.72E-18	1.43E-15
**IRGM**	2.272557372	-2.634843472	2.04E-18	1.63E-15
**SPRR2E**	3.827456634	0.205928492	5.86E-18	4.46E-15
**IGF2**	2.563406708	10.49892132	7.75E-18	5.66E-15
**SFTPA1**	-3.439079681	-0.478289005	8.42E-18	5.91E-15
**MYBPC3**	-1.48711947	-0.72412177	1.04E-17	7.03E-15
**CHRND**	2.779526746	-2.042282053	1.68E-17	1.08E-14
**CPA1**	-3.415027205	-1.881128102	1.73E-17	1.08E-14
**LRP1B**	2.14079152	-1.579753304	3.92E-17	2.37E-14
**FGF6**	2.91374155	-3.712582017	6.94E-17	3.92E-14
**NCAN**	2.069471865	-2.591667918	7.81E-17	4.28E-14
**CRNN**	3.121376294	-3.60935195	8.49E-17	4.51E-14
**TEX19**	1.666377162	-1.836186616	9.36E-17	4.83E-14
**KRT78**	2.822613577	-2.021934903	1.13E-16	5.57E-14
**SPRR2B**	3.129010161	-3.678003165	1.14E-16	5.57E-14
**CER1**	2.520803791	-2.203849013	1.47E-16	6.85E-14
**HSF5**	-1.99545602	-2.612251274	1.48E-16	6.85E-14
**PSCA**	-2.380693502	2.753820518	1.73E-16	7.79E-14
**SOHLH1**	3.217355233	-0.87598646	1.96E-16	8.59E-14
**TCAP**	-1.342674905	0.49314832	2.07E-16	8.85E-14
**CACNG6**	2.571855257	-2.348208956	2.26E-16	9.44E-14
**PPP1R17**	2.936777338	-3.121890288	2.53E-16	1.03E-13

**LCE3D**	3.16397936	-3.504363046	5.08E-16	2.02E-13
**ALK**	-2.076681305	-1.875248666	5.62E-16	2.19E-13
**BTBD18**	-1.748492802	-2.427795621	6.52E-16	2.45E-13
**NLRP4**	2.427579094	-2.462632298	6.58E-16	2.45E-13
**COX8C**	2.486207698	-3.403147774	7.59E-16	2.77E-13
**UNCX**	3.181024653	-2.975161822	1.86E-15	6.51E-13
**PAX2**	1.931472173	-2.011428735	1.96E-15	6.75E-13
**PRSS56**	3.0779817	2.524854341	2.50E-15	8.44E-13
**ADAD2**	-2.074390989	-2.246377052	3.51E-15	1.16E-12
**KRTAP4-6**	3.125411344	-3.474655447	9.14E-15	2.91E-12
**AIRE**	1.96966077	0.107194373	1.53E-14	4.72E-12
**GPR50**	2.78225342	-3.512308452	1.95E-14	5.90E-12
**DHRS9**	-1.388689122	4.241384982	2.94E-14	8.73E-12
**CTRB1**	-2.843935669	-3.285432994	3.48E-14	1.02E-11
**PNLIP**	-4.197716375	-2.264222644	3.56E-14	1.02E-11
**CHST8**	1.797092714	-2.300392537	5.16E-14	1.46E-11
**INHBB**	1.147711395	3.368747483	5.47E-14	1.50E-11
**OR1N2**	3.024651126	-3.356161211	6.49E-14	1.75E-11
**SP8**	1.769885776	0.470026551	7.22E-14	1.92E-11
**TCHH**	1.555387264	0.242670279	1.07E-13	2.80E-11
**WIPF3**	-1.266558754	1.550177233	1.28E-13	3.25E-11
**SBK2**	2.646050718	-3.338877219	1.71E-13	4.23E-11
**CNBD2**	-1.2221705	-1.135123687	1.72E-13	4.23E-11
**KCNJ9**	1.438795994	-2.338008577	1.74E-13	4.23E-11
**HCN4**	1.531837305	-2.65326836	2.47E-13	5.94E-11
**CELA2A**	-2.365614471	-3.108354642	2.86E-13	6.78E-11
**HRH3**	2.446853002	-2.597516531	3.55E-13	8.30E-11
**CLCA4**	-1.904798845	4.992553007	3.86E-13	8.89E-11
**PNMT**	-1.612884443	-0.339086253	3.97E-13	9.03E-11
**NEUROD2**	1.759126895	-2.333313622	4.08E-13	9.17E-11
**MMP8**	-1.961985253	0.571850369	4.37E-13	9.70E-11
**PAK5**	2.841729235	-1.772739671	4.60E-13	9.98E-11
**CRCT1**	2.981378717	-2.212261362	4.61E-13	9.98E-11
**OPRD1**	1.476703756	0.695543527	7.70E-13	1.58E-10
**DHRS2**	1.332597941	2.501048869	7.94E-13	1.59E-10
**CTRB2**	-2.339125468	-3.115584479	8.19E-13	1.59E-10
**SHISA7**	1.626093487	-2.647678966	9.57E-13	1.84E-10
**CYP3A4**	-1.520113957	0.482015723	1.27E-12	2.42E-10
**SLC8A3**	-1.379487399	-1.628510541	1.30E-12	2.45E-10
**KCNQ2**	1.974924013	-0.044041484	1.36E-12	2.54E-10
**CELA3A**	-1.94394047	-2.217306856	2.54E-12	4.55E-10
**TMCO2**	-1.945719141	-3.354927364	3.05E-12	5.41E-10
**NFE4**	-1.739892722	-2.877466698	3.36E-12	5.89E-10
**PDYN**	2.560031059	-3.636925717	3.44E-12	5.96E-10

**BARX1**	1.691389229	-0.259556361	5.21E-12	8.62E-10
**CHRNA2**	2.042761335	-3.190945488	6.43E-12	1.03E-09
**SLC1A6**	1.834970028	-3.434209537	8.12E-12	1.29E-09
**TDRD12**	-1.237054104	-1.516326361	8.24E-12	1.29E-09
**CD70**	-1.193851493	1.432471366	9.15E-12	1.41E-09
**ABCB11**	-1.270773532	-2.107297794	9.22E-12	1.41E-09
**PRSS41**	1.456611503	-0.983107083	1.01E-11	1.51E-09
**KRT14**	2.720442947	2.355297442	1.11E-11	1.63E-09
**CDH22**	1.984282904	-1.498088471	1.12E-11	1.63E-09
**CELF3**	1.334198828	1.34714077	1.21E-11	1.74E-09
**SEZ6**	1.605158147	-1.428244034	1.23E-11	1.76E-09
**DLX2**	1.456959662	-1.531068336	1.45E-11	1.99E-09
**TMPRSS5**	1.188163331	1.215840547	1.57E-11	2.13E-09
**PRH2**	1.44047523	-2.489834284	2.09E-11	2.71E-09
**TCP11**	1.958915549	0.628046368	2.30E-11	2.96E-09
**SERPINB7**	-1.879016888	0.650112055	2.89E-11	3.67E-09
**PNLDC1**	1.342523085	-1.597788641	3.07E-11	3.87E-09
**DDX53**	-3.147571219	-2.41742091	3.14E-11	3.94E-09
**RGR**	1.773931954	-1.055324704	3.99E-11	4.85E-09
**RNF17**	1.665438007	-3.143353885	4.60E-11	5.45E-09
**ARHGAP36**	1.889941336	-2.657382982	4.69E-11	5.51E-09
**ATP2B3**	1.70121118	-2.618004557	7.48E-11	8.46E-09
**SEC14L4**	1.556686352	-0.377906383	7.91E-11	8.89E-09
**ERVW-1**	-1.376610531	-1.581971543	8.04E-11	8.98E-09
**KRT7**	-1.209797366	4.110393537	8.90E-11	9.87E-09
**KRT6C**	1.948661231	-1.072132021	9.09E-11	1.00E-08
**CACNA2D2**	1.025266962	2.907545249	9.84E-11	1.07E-08
**SRRM4**	1.765300976	-2.728533085	1.17E-10	1.24E-08
**MAGEB1**	-3.028804539	-3.717088833	1.28E-10	1.36E-08
**UMODL1**	1.112933414	0.717474722	1.35E-10	1.41E-08
**SLITRK6**	1.489366187	3.640262314	1.42E-10	1.49E-08
**FBN3**	1.505139225	-2.368332795	1.50E-10	1.53E-08
**SPRR3**	2.385699135	1.429251748	1.72E-10	1.71E-08
**CCDC141**	-1.115074419	-1.294554774	2.72E-10	2.62E-08
**CACNG7**	1.67991275	-2.720958717	3.11E-10	2.93E-08
**GRIN2C**	1.015413002	0.66198504	3.67E-10	3.39E-08
**EEF1A2**	1.291485377	1.582386617	3.84E-10	3.53E-08
**CPNE6**	1.250244443	-0.167454221	3.93E-10	3.58E-08
**P2RY4**	-1.216599884	-2.623647067	4.51E-10	4.06E-08
**CAPN6**	1.340517885	4.041772872	4.99E-10	4.43E-08
**ALPP**	-1.64562261	-0.096982685	5.00E-10	4.43E-08
**TMEM249**	1.075664405	-0.134121212	5.22E-10	4.57E-08
**CLCA2**	1.706035925	0.32499038	6.13E-10	5.32E-08
**PPBP**	-2.170318006	6.34585552	7.29E-10	6.26E-08

**PCSK2**	1.592723522	-1.608734244	9.60E-10	8.01E-08
**RIMS2**	-1.666060074	-1.222038836	9.79E-10	8.14E-08
**SPRR2D**	1.959262185	0.956288191	1.06E-09	8.76E-08
**SERPINB13**	2.736705482	-2.058738202	1.18E-09	9.64E-08
**AC011473.4**	2.091699385	-3.587649334	1.46E-09	1.15E-07
**ACTL6B**	1.466350588	-3.249490202	1.47E-09	1.15E-07
**HMX2**	1.6543069	-2.435922227	1.47E-09	1.15E-07
**CLDN6**	-1.562265872	-0.891066946	1.50E-09	1.17E-07
**RHBDL3**	1.14009119	0.434193314	1.64E-09	1.26E-07
**UGT2B4**	2.173370275	-0.20174877	2.02E-09	1.49E-07
**CYP4F8**	1.298865172	-0.629100978	2.27E-09	1.64E-07
**NDST4**	1.845492741	-3.175543407	2.55E-09	1.80E-07
**NKX2-2**	1.583254033	-1.601034935	2.55E-09	1.80E-07
**INSL4**	2.413989629	-1.175278026	2.68E-09	1.86E-07
**CALML5**	-2.273861934	-1.992306365	2.81E-09	1.94E-07
**C5orf58**	-1.037876506	-1.735089575	2.92E-09	2.01E-07
**IL36G**	1.222289947	-1.891129026	2.99E-09	2.05E-07
**ANPEP**	-1.155797369	7.161288308	3.36E-09	2.24E-07
**GCGR**	1.177633758	-2.25130132	3.57E-09	2.37E-07
**CIDEC**	-1.006543467	1.671178547	3.66E-09	2.42E-07
**RS1**	1.25955313	-3.054273066	3.68E-09	2.42E-07
**FOXI1**	1.682684633	-0.479592005	4.83E-09	3.08E-07
**LEFTY2**	1.148378372	0.353095606	4.89E-09	3.10E-07
**SCRT1**	1.280838422	-2.617093698	5.85E-09	3.66E-07
**GLYATL2**	-1.228849147	-1.349026152	6.34E-09	3.90E-07
**GPR87**	-1.948147881	-1.890206479	6.45E-09	3.95E-07
**INSRR**	1.063315874	-2.445268642	7.38E-09	4.43E-07
**NTNG1**	-1.300477194	-1.722775507	7.44E-09	4.43E-07
**ADAM2**	1.777040571	-3.335065132	7.71E-09	4.55E-07
**ALDOB**	-1.025146468	5.547756919	7.93E-09	4.62E-07
**MEGF11**	1.041476981	0.386090685	8.00E-09	4.64E-07
**CELA3B**	-1.434188142	-2.383918049	8.03E-09	4.64E-07
**C20orf85**	1.739171978	-2.502521014	8.15E-09	4.70E-07
**VWA5B2**	1.095715836	0.466411695	8.25E-09	4.74E-07
**LEFTY1**	1.155277139	6.321533592	9.19E-09	5.16E-07
**NAT8L**	1.084865481	0.505084139	9.97E-09	5.57E-07
**CCDC155**	1.427208279	-2.874974706	1.05E-08	5.83E-07
**ITLN2**	1.679096063	0.6358475	1.13E-08	6.13E-07
**LRRTM1**	1.480192499	0.252495947	1.20E-08	6.47E-07
**BMP3**	-1.1499052	0.914480767	1.32E-08	7.04E-07
**KIF19**	1.149967323	1.847846877	1.50E-08	7.85E-07
**FGF21**	1.331636376	-3.470099748	1.71E-08	8.85E-07
**QRFPR**	-1.736711044	-1.734904854	2.06E-08	1.04E-06
**PHF21B**	1.339902143	-3.122491513	2.43E-08	1.20E-06

**TAS2R30**	-1.613823866	-2.866099183	2.44E-08	1.20E-06
**USP41**	1.027094403	-2.051916019	2.48E-08	1.21E-06
**KRT76**	-2.257514856	-3.563908016	3.02E-08	1.43E-06
**CPS1**	1.436976038	4.42241397	3.29E-08	1.54E-06
**PCDHA1**	1.523009509	-2.146860766	3.52E-08	1.62E-06
**ANXA8L1**	-1.072530351	-1.787954743	3.70E-08	1.69E-06
**PTH2R**	1.575880566	-1.298939013	3.88E-08	1.76E-06
**SPRR2A**	1.829327248	0.946647399	4.06E-08	1.82E-06
**SLC26A3**	-1.179878857	7.406954368	4.08E-08	1.83E-06
**SEMG2**	-1.940789397	-1.821519076	4.13E-08	1.84E-06
**PSG9**	-2.456744059	-3.248984906	4.52E-08	2.00E-06
**A2ML1**	-1.612475068	0.316025451	4.53E-08	2.00E-06
**SYNGR4**	-1.192763413	0.071144104	5.01E-08	2.17E-06
**HRG**	1.441570096	-3.119608232	5.42E-08	2.34E-06
**AC010422.3**	1.032795565	1.905839048	5.46E-08	2.34E-06
**CNMD**	-1.291103929	-3.310846572	5.49E-08	2.35E-06
**ADGRA1**	1.454558391	-3.421129509	5.55E-08	2.36E-06
**SH3GL2**	1.235520063	-1.193773724	7.08E-08	2.89E-06
**EPHA7**	1.071642448	1.248143004	7.09E-08	2.89E-06
**MLN**	-2.010778002	-2.279278042	7.44E-08	3.00E-06
**HTR2C**	2.087913234	-2.335425318	7.53E-08	3.02E-06
**KRT24**	2.002689731	-2.678487359	7.77E-08	3.07E-06
**DPYSL5**	1.356810903	-1.59769035	7.77E-08	3.07E-06
**SFTPD**	-1.06092138	-2.287668046	7.96E-08	3.14E-06
**FAM83A**	-1.066072008	1.642678363	8.86E-08	3.41E-06
**ADGRD2**	1.097729983	-2.357265801	8.90E-08	3.42E-06
**HPN**	1.230507478	2.315805719	9.17E-08	3.49E-06
**ZFR2**	1.125197101	-2.634539878	9.36E-08	3.54E-06
**ORM1**	-1.825574524	1.776117953	1.08E-07	4.01E-06
**PLSCR5**	-2.229568086	-3.616716022	1.09E-07	4.01E-06
**SCRT2**	1.662829435	-3.571194228	1.32E-07	4.73E-06
**LCN6**	-1.104442253	-2.719957558	1.57E-07	5.49E-06
**FAM9C**	-1.145494933	-3.422728219	1.70E-07	5.80E-06
**LIPC**	1.218534769	0.66502447	1.98E-07	6.60E-06
**KRT6A**	1.51171034	3.669255108	1.98E-07	6.60E-06
**AMY2A**	-1.639029008	-3.462558581	2.15E-07	7.07E-06
**CDH16**	-1.336133231	1.499704389	2.74E-07	8.59E-06
**REG3A**	1.601378841	7.995142696	3.28E-07	1.01E-05
**DLX1**	1.183074417	-1.113477782	3.52E-07	1.07E-05
**CA6**	1.590979932	-1.559318578	3.52E-07	1.07E-05
**LMX1A**	1.377630741	-2.182366956	3.61E-07	1.09E-05
**PCOLCE2**	-1.027148657	0.554127664	3.80E-07	1.13E-05
**GNG13**	1.075195125	-1.246392158	3.89E-07	1.15E-05
**IVL**	1.935794139	-1.278514244	3.90E-07	1.16E-05

**IL1RAPL2**	-1.71926242	-2.880461471	3.98E-07	1.17E-05
**FOXN4**	1.222937243	-3.310641981	4.24E-07	1.24E-05
**COX7B2**	-1.966537569	-3.111071188	4.61E-07	1.34E-05
**GPR22**	-1.153278497	-2.279866749	4.69E-07	1.35E-05
**LRTM2**	1.052173957	-3.411412176	5.09E-07	1.46E-05
**PON1**	-1.278757495	-0.945240411	5.16E-07	1.47E-05
**IRX4**	-1.718776348	-3.116241545	5.60E-07	1.57E-05
**SCGB3A2**	-1.441865854	-3.026701648	5.69E-07	1.59E-05
**SERPINA11**	1.378179449	-1.811214954	6.01E-07	1.66E-05
**LKAAEAR1**	1.331958674	-3.37368198	6.30E-07	1.72E-05
**GPR83**	1.1770591	-0.269450184	6.69E-07	1.81E-05
**SCEL**	-1.088131856	1.697057034	6.93E-07	1.86E-05
**AKAIN1**	1.403897457	-2.437698672	7.03E-07	1.87E-05
**HTR3E**	1.135562809	-2.10531703	7.56E-07	2.00E-05
**CNGA3**	-1.193455476	-0.477960055	7.61E-07	2.01E-05
**HTR3C**	1.488664513	-2.229110873	8.66E-07	2.23E-05
**RGS7**	1.194365512	-1.757266771	9.09E-07	2.32E-05
**BRDT**	-1.156148158	-3.457943499	9.96E-07	2.51E-05
**APOA2**	1.490466446	0.106074343	1.13E-06	2.78E-05
**TMIGD1**	-1.316058297	0.851301429	1.17E-06	2.85E-05
**TAS2R46**	-1.228466891	-3.488484295	1.23E-06	2.96E-05
**F7**	1.102001808	1.457242184	1.30E-06	3.10E-05
**RETNLB**	1.080975389	4.277457507	1.31E-06	3.11E-05
**TCF23**	1.20021154	-1.655390852	1.66E-06	3.83E-05
**SI**	-1.084575822	4.016751472	1.69E-06	3.87E-05
**MROH2A**	1.235634632	-2.814313256	1.71E-06	3.90E-05
**DBH**	-1.018779801	-0.434679727	1.73E-06	3.93E-05
**IL36B**	-1.485255972	-2.164042876	1.77E-06	4.01E-05
**TMPRSS11E**	1.585345467	-0.232020961	1.92E-06	4.29E-05
**SLC18A1**	1.059510597	1.895254921	2.08E-06	4.57E-05
**CBLN4**	1.265128306	-1.765051863	2.28E-06	4.97E-05
**SYNPR**	1.198667481	-0.251976384	2.32E-06	5.04E-05
**SIX2**	1.024686629	0.952835916	2.36E-06	5.11E-05
**SLC7A10**	1.193148778	-1.212881712	2.66E-06	5.59E-05
**KRT31**	-1.596779565	-1.612257678	2.73E-06	5.71E-05
**CALB1**	-1.469845281	3.888137885	2.77E-06	5.76E-05
**GABRA4**	1.173149584	0.558600712	2.86E-06	5.90E-05
**LIPF**	1.732069425	-2.941925123	3.02E-06	6.15E-05
**RFX6**	1.25369584	-0.634922452	3.46E-06	6.91E-05
**GHRH**	1.179160298	-3.433510976	3.82E-06	7.46E-05
**NOL4**	1.058766961	-1.1998089	4.31E-06	8.25E-05
**KLK4**	1.028265065	0.35637354	4.48E-06	8.51E-05
**AQP8**	-1.144743706	3.247293326	5.06E-06	9.37E-05
**CHGB**	1.200485926	2.836162831	5.69E-06	0.000103233

**KIF1A**	1.040059092	0.941348924	5.83E-06	0.000104919
**AKAP4**	-1.159291566	-1.976167345	6.01E-06	0.000107804
**ANKRD34C**	1.247375821	-3.589733934	6.72E-06	0.000118862
**CACNG4**	-1.063727225	2.336925204	6.82E-06	0.000120246
**SOHLH2**	-1.419136705	-2.070169281	7.06E-06	0.00012386
**MAGEB2**	-1.992414241	-0.579434297	7.28E-06	0.000126893
**KLK13**	1.067034787	-0.51282841	7.64E-06	0.000132212
**SLITRK1**	1.410744984	-3.581544674	7.82E-06	0.000134392
**ALPPL2**	-1.190984371	2.47789863	8.45E-06	0.00014381
**ATP6V0A4**	1.055196816	-1.414245216	9.02E-06	0.000151421
**KRT9**	-1.012319367	-3.238618065	9.22E-06	0.000153974
**MUC15**	1.456966054	-2.219333137	9.68E-06	0.000159845
**XAGE2**	-1.808962879	-1.808414008	1.01E-05	0.000164496
**POU3F3**	1.048796203	-1.86806727	1.07E-05	0.000172582
**CNTN5**	1.187266099	-2.589475135	1.12E-05	0.00017861
**GFY**	1.155077302	-3.13489963	1.34E-05	0.000206705
**HIST1H4C**	-1.487047722	2.253037179	1.37E-05	0.000209621
**TRPV6**	-1.13485009	0.899081358	1.41E-05	0.000215684
**UPK2**	-1.077626176	-0.743070621	1.42E-05	0.000216651
**HS3ST4**	1.418823059	-0.235006914	1.44E-05	0.000218624
**ACTBL2**	-1.144936111	-2.52228056	1.47E-05	0.000221517
**SLCO1A2**	1.06392193	-1.217702555	1.47E-05	0.000221833
**SLC6A2**	1.41483817	-2.222592277	1.47E-05	0.000222007
**TMPRSS11D**	1.289941605	-3.083001116	1.56E-05	0.000231348
**REG3G**	-1.658377924	-0.065033224	1.58E-05	0.000234379
**NEUROD1**	1.521181383	-0.159254131	1.84E-05	0.000263949
**TBX4**	1.103203816	0.275787257	1.84E-05	0.000264279
**ZPBP2**	-1.12558078	-3.118840938	1.85E-05	0.000264824
**KCNC2**	1.3914501	-2.467029689	1.97E-05	0.000278433
**BPIFB4**	1.36307	-3.484054024	2.34E-05	0.000319043
**SLC10A2**	-1.652554517	-2.685726753	2.39E-05	0.000323862
**SLURP1**	1.241910514	-3.351437732	2.46E-05	0.000332226
**CA1**	-1.125328837	3.284431918	2.54E-05	0.000340671
**PCSK1**	1.065772313	5.086658269	2.65E-05	0.000351976
**TUBA3C**	-1.678644007	-3.283817168	2.75E-05	0.00036331
**SLC38A8**	1.160550364	-2.669416324	2.75E-05	0.00036331
**ANKRD34B**	1.149465206	-3.024732677	2.77E-05	0.000364455
**MYBPHL**	1.118876875	-0.558534906	2.84E-05	0.000370229
**MAGEC1**	-1.766799968	-2.235964611	3.02E-05	0.000391743
**SP9**	1.476159349	-1.499783862	3.09E-05	0.000397231
**SLC30A10**	-1.002782647	-1.089751727	3.14E-05	0.000401186
**CLPS**	-1.561650033	-2.981534836	3.32E-05	0.000421658
**SPRR1A**	1.408086339	0.84222544	3.50E-05	0.000437299
**RPTN**	-1.604655949	-2.843601984	3.59E-05	0.000445504

**NKX2-5**	1.197036039	-2.708422535	3.72E-05	0.000457521
**SFTPB**	-1.197280462	0.722946789	3.72E-05	0.000457521
**SLC36A2**	1.053655149	-3.57386111	3.79E-05	0.000465843
**KCNE1B**	-1.529444356	-1.380753954	4.14E-05	0.000496502
**ANXA8**	-1.217741801	-1.512656003	4.15E-05	0.00049755
**MS4A12**	-1.06152352	3.317541034	4.38E-05	0.000519716
**NHLH2**	1.356809635	-1.602425103	4.38E-05	0.000519716
**GSX1**	1.156607961	-3.439566749	4.41E-05	0.000522765
**TKTL1**	1.391449925	0.893977857	4.43E-05	0.000523732
**HSD3B2**	-1.143277422	-0.775882722	4.50E-05	0.000529268
**INSL5**	-1.312826278	-1.136401805	4.65E-05	0.000545304
**GDF10**	1.159520598	1.127218941	4.67E-05	0.000546804
**DAZ1**	1.816757088	-3.46557901	5.16E-05	0.000594926
**KRT13**	1.024830918	-0.050267276	5.20E-05	0.000597986
**KRT5**	1.307461575	3.752473133	5.47E-05	0.000625891
**CNPY1**	1.130801145	-3.252149028	5.52E-05	0.000629496
**CRISP3**	-1.474739329	-3.326274713	5.59E-05	0.000636181
**CCDC190**	1.30570221	-1.716021437	5.65E-05	0.000640593
**PAX7**	1.583026048	-0.537417348	6.00E-05	0.000674102
**FOXR2**	1.544757242	-3.669269745	6.17E-05	0.00069018
**LCN15**	1.244454742	5.656950865	6.97E-05	0.000759527
**MUC16**	-1.069871046	1.225839054	7.54E-05	0.000806974
**DMRT1**	1.270752307	-1.710486211	7.94E-05	0.000840587
**IFNK**	-1.286961629	-3.654647063	7.97E-05	0.000842582
**HIST1H1B**	-1.29125851	3.281342721	8.78E-05	0.000906862
**POU5F2**	-1.015630983	-2.679188817	8.78E-05	0.000906862
**CLDN8**	-1.28242253	0.198302867	9.32E-05	0.000954424
**HIST1H4D**	-1.260000086	1.80701295	9.67E-05	0.000984683
**NTS**	-1.254919642	2.513937584	9.72E-05	0.000988852
**BRINP3**	1.147682142	0.576712819	0.000108159	0.001077285
**KRTAP13-2**	-1.272705137	-3.339338189	0.000115078	0.001137161
**GC**	1.301862444	-3.173059503	0.000116538	0.001148989
**PSAPL1**	-1.294235336	-1.564879962	0.000120085	0.001174711
**HIST1H4F**	1.569104577	-1.831521911	0.000123732	0.001203011
**LCN8**	1.098985645	-2.543127272	0.000164142	0.001517625
**TBX5**	1.206332606	-2.004452939	0.00016708	0.001535874
**CPB1**	-1.157551951	-1.571661647	0.00018106	0.001631867
**HIST1H2BO**	-1.090138805	0.914031325	0.000218915	0.001892294
**CT45A1**	-1.649937937	-2.976959656	0.000220593	0.001903051
**TRIM71**	1.051241338	-0.186310634	0.000232016	0.00198209
**LRRC53**	1.160724897	-2.964498748	0.000233996	0.001991598
**11-Mar**	1.087333495	-3.592032111	0.000252393	0.002109897
**PAGE2**	1.080463915	-3.577203944	0.000291123	0.002370487
**SSX1**	-1.647567942	-3.460640374	0.000293042	0.00238156

**REG1B**	1.19452104	6.927395912	0.000293741	0.002386132
**TEX15**	-1.159977928	-3.091763936	0.000308843	0.002486918
**ZIC4**	-1.396251346	-2.764807107	0.00038686	0.00296401
**PIP**	1.010729367	-3.424654191	0.000393514	0.003006771
**CLPSL1**	-1.377399839	-3.301128892	0.00042546	0.003196878
**PNMA5**	1.441611028	2.630083872	0.000444708	0.003311695
**DCAF4L2**	-1.583191875	-2.711888476	0.000456197	0.00338575
**OTOP3**	-1.199681696	-3.241997401	0.000526444	0.003771378
**HIST1H1D**	-1.063336504	1.884717849	0.000576363	0.004074048
**SOX21**	-1.016395538	-2.701560782	0.000582663	0.004110297
**HIST1H1A**	-1.339973526	-3.524797392	0.000614555	0.004302378
**SSX5**	1.304791988	-3.509225395	0.000714199	0.004840448
**HAND1**	-1.007689624	0.150128226	0.000740713	0.004973074
**PTF1A**	-1.160166067	-1.125245819	0.000743644	0.004985115
**SAGE1**	1.146086463	-2.513457428	0.001821466	0.009910085
**FGL1**	1.021462337	-0.750700965	0.002317434	0.011969541
**TAAR1**	-1.114927363	-3.437069014	0.003324611	0.015923615
**MAGEA12**	1.294130666	1.187904434	0.00343474	0.016312923
**CSAG3**	1.022079497	-2.546316893	0.005409409	0.023117243
**CSAG1**	1.081832341	0.35954189	0.006358055	0.026188137
**SFTA3**	-1.161041651	-1.800515598	0.006766066	0.027462178
**MT4**	-1.066899531	-0.882531009	0.008054238	0.031292284
**CXorf67**	-1.049342469	-3.004583696	0.010746722	0.039020307
**SPRR2F**	1.058365605	-1.095205669	0.013989163	0.047654494
**MAGEA9**	1.01509734	-3.594548206	0.014354068	0.048638898

Table S7. Survival analysis results of DEG between high - and low -AC096751.1 phenotypes in COAD OS (Only gene list with P<0.05 is shown).

ID	P	HR	Low 95%CI	High 95%CI
**COX8C**	0.003675859	1.860622743	1.223786796	2.828856301
**PNMA5**	0.007848828	1.777282954	1.163122031	2.715738001
**PCOLCE2**	0.013690667	1.711254184	1.116408425	2.623046206
**TAS2R30**	0.01442023	1.691660366	1.110232037	2.577582613
**AQP8**	0.017066227	0.60147528	0.396098115	0.913340656
**COLEC10**	0.018999405	0.604562301	0.397018326	0.920601272
**EPHA7**	0.019309313	0.605979641	0.39829806	0.921951077
**SLC38A8**	0.019959953	1.635028911	1.080654648	2.473796363
**ALPP**	0.020135118	1.648264896	1.081387651	2.512306447
**ITLN2**	0.02052065	0.61121153	0.403000384	0.92699548
**GCG**	0.021026946	0.613833632	0.405530371	0.929133191
**DPCR1**	0.023376864	1.631880162	1.068620831	2.492027842
**GLYATL2**	0.023987821	0.621798967	0.411606996	0.93932795
**GFY**	0.026389779	1.601463781	1.056794432	2.426854424
**PNLDC1**	0.026596309	1.610906349	1.056914423	2.455278506
**GPR83**	0.03274764	1.579881157	1.038240632	2.404090527
**PCSK1**	0.03710554	0.642766157	0.424211455	0.973920737
**ZFR2**	0.046216879	1.52394574	1.007093193	2.306053337

Table S8. Functional enrichment analysis results of DEG between high - and low -AC096751.1 phenotypes in COAD

Category	Term	Count	PValue
**GOTERM_CC_DIRECT**	GO:0005576~extracellular region	73	4.22E-12
**GOTERM_CC_DIRECT**	GO:0005615~extracellular space	64	1.90E-11
**GOTERM_CC_DIRECT**	GO:0001533~cornified envelope	12	7.27E-10
**GOTERM_BP_DIRECT**	GO:0008544~epidermis development	15	8.63E-10
**GOTERM_MF_DIRECT**	GO:0005198~structural molecule activity	22	8.99E-09
**GOTERM_BP_DIRECT**	GO:0031424~keratinization	10	2.95E-07
**GOTERM_BP_DIRECT**	GO:0018149~peptide cross-linking	10	4.25E-07
**GOTERM_MF_DIRECT**	GO:0004252~serine-type endopeptidase activity	19	1.64E-06
**GOTERM_BP_DIRECT**	GO:0030216~keratinocyte differentiation	11	1.98E-06
**GOTERM_CC_DIRECT**	GO:0045334~clathrin-coated endocytic vesicle	6	6.15E-06
**GOTERM_CC_DIRECT**	GO:0042599~lamellar body	5	8.42E-06
**GOTERM_MF_DIRECT**	GO:0005179~hormone activity	11	1.06E-05
**GOTERM_BP_DIRECT**	GO:0006508~proteolysis	26	1.53E-05
**GOTERM_BP_DIRECT**	GO:0010842~retina layer formation	6	5.29E-05
**GOTERM_CC_DIRECT**	GO:0005882~intermediate filament	11	5.87E-05
**GOTERM_BP_DIRECT**	GO:0035881~amacrine cell differentiation	4	6.98E-05
**GOTERM_MF_DIRECT**	GO:0001228~transcriptional activator activity, RNA polymerase II transcription regulatory region sequence-specific binding	10	7.39E-05
**GOTERM_BP_DIRECT**	GO:0007585~respiratory gaseous exchange	6	4.62E-04
**GOTERM_BP_DIRECT**	GO:0006810~transport	18	4.64E-04
**GOTERM_BP_DIRECT**	GO:0044267~cellular protein metabolic process	10	4.81E-04
**GOTERM_CC_DIRECT**	GO:0045095~keratin filament	9	6.36E-04
**GOTERM_BP_DIRECT**	GO:0007271~synaptic transmission, cholinergic	6	6.90E-04
**GOTERM_MF_DIRECT**	GO:0015464~acetylcholine receptor activity	5	8.15E-04
**GOTERM_MF_DIRECT**	GO:0004889~acetylcholine-activated cation-selective channel activity	5	8.15E-04
**GOTERM_CC_DIRECT**	GO:0005887~integral component of plasma membrane	45	8.22E-04
**GOTERM_CC_DIRECT**	GO:0005892~acetylcholine-gated channel complex	5	0.001005

**GOTERM_MF_DIRECT**	GO:0008236~serine-type peptidase activity	7	0.001156
**GOTERM_BP_DIRECT**	GO:0007626~locomotory behavior	8	0.001207
**GOTERM_CC_DIRECT**	GO:0034774~secretory granule lumen	4	0.001314
**GOTERM_MF_DIRECT**	GO:0042166~acetylcholine binding	5	0.001316
**GOTERM_MF_DIRECT**	GO:0005125~cytokine activity	11	0.00184
**GOTERM_MF_DIRECT**	GO:0000977~RNA polymerase II regulatory region sequence-specific DNA binding	12	0.001961
**GOTERM_BP_DIRECT**	GO:0098655~cation transmembrane transport	6	0.00228
**GOTERM_BP_DIRECT**	GO:0007417~central nervous system development	9	0.002326
**GOTERM_MF_DIRECT**	GO:0005230~extracellular ligand-gated ion channel activity	5	0.003249
**GOTERM_BP_DIRECT**	GO:0042391~regulation of membrane potential	7	0.003297
**GOTERM_BP_DIRECT**	GO:0007267~cell-cell signaling	13	0.00405
**GOTERM_MF_DIRECT**	GO:0015276~ligand-gated ion channel activity	5	0.004476
**GOTERM_BP_DIRECT**	GO:0071377~cellular response to glucagon stimulus	5	0.007274
**GOTERM_BP_DIRECT**	GO:0048468~cell development	5	0.007274
**GOTERM_BP_DIRECT**	GO:0032094~response to food	4	0.007377
**GOTERM_MF_DIRECT**	GO:0005160~transforming growth factor beta receptor binding	5	0.007791
**GOTERM_BP_DIRECT**	GO:0043408~regulation of MAPK cascade	5	0.00794
**GOTERM_BP_DIRECT**	GO:0030154~cell differentiation	18	0.00885
**GOTERM_CC_DIRECT**	GO:0000786~nucleosome	7	0.00902
**GOTERM_BP_DIRECT**	GO:0050796~regulation of insulin secretion	6	0.009557
**GOTERM_MF_DIRECT**	GO:0008083~growth factor activity	9	0.011585
**GOTERM_BP_DIRECT**	GO:0006811~ion transport	8	0.011826
**GOTERM_BP_DIRECT**	GO:0010862~positive regulation of pathway-restricted SMAD protein phosphorylation	5	0.013734
**GOTERM_CC_DIRECT**	GO:0005891~voltage-gated calcium channel complex	4	0.017207
**GOTERM_MF_DIRECT**	GO:0005200~structural constituent of cytoskeleton	7	0.017592
**GOTERM_BP_DIRECT**	GO:0071320~cellular response to cAMP	5	0.017999
**GOTERM_BP_DIRECT**	GO:0007283~spermatogenesis	15	0.018178

**GOTERM_BP_DIRECT**	GO:0007268~chemical synaptic transmission	11	0.018789
**GOTERM_CC_DIRECT**	GO:0031093~platelet alpha granule lumen	5	0.020385
**GOTERM_BP_DIRECT**	GO:0043085~positive regulation of catalytic activity	6	0.02041
**GOTERM_MF_DIRECT**	GO:0046983~protein dimerization activity	8	0.02329
**GOTERM_MF_DIRECT**	GO:0005249~voltage-gated potassium channel activity	5	0.02341
**GOTERM_BP_DIRECT**	GO:0003007~heart morphogenesis	4	0.023543
**GOTERM_BP_DIRECT**	GO:0007210~serotonin receptor signaling pathway	3	0.025303
**GOTERM_BP_DIRECT**	GO:0021527~spinal cord association neuron differentiation	3	0.025303
**GOTERM_BP_DIRECT**	GO:0044241~lipid digestion	3	0.025303
**GOTERM_BP_DIRECT**	GO:0070588~calcium ion transmembrane transport	7	0.02823
**GOTERM_BP_DIRECT**	GO:0006334~nucleosome assembly	7	0.02823
**GOTERM_BP_DIRECT**	GO:0002009~morphogenesis of an epithelium	3	0.029148
**GOTERM_CC_DIRECT**	GO:0043195~terminal bouton	5	0.030104
**GOTERM_BP_DIRECT**	GO:0060395~SMAD protein signal transduction	5	0.031961
**GOTERM_MF_DIRECT**	GO:0030246~carbohydrate binding	9	0.032009
**GOTERM_BP_DIRECT**	GO:0009888~tissue development	3	0.03321
**GOTERM_BP_DIRECT**	GO:0007586~digestion	5	0.033626
**GOTERM_MF_DIRECT**	GO:0043565~sequence-specific DNA binding	17	0.036029
**GOTERM_BP_DIRECT**	GO:0021893~cerebral cortex GABAergic interneuron fate commitment	2	0.038335
**GOTERM_BP_DIRECT**	GO:0003218~cardiac left ventricle formation	2	0.038335
**GOTERM_BP_DIRECT**	GO:0003326~pancreatic A cell fate commitment	2	0.038335
**GOTERM_BP_DIRECT**	GO:0003329~pancreatic PP cell fate commitment	2	0.038335
**GOTERM_MF_DIRECT**	GO:0005245~voltage-gated calcium channel activity	4	0.03888
**GOTERM_MF_DIRECT**	GO:0004806~triglyceride lipase activity	3	0.039705
**GOTERM_BP_DIRECT**	GO:0006366~transcription from RNA polymerase II promoter	17	0.041449
**GOTERM_BP_DIRECT**	GO:0001580~detection of chemical stimulus involved in sensory perception of bitter taste	4	0.041919
**GOTERM_BP_DIRECT**	GO:0030326~embryonic limb morphogenesis	4	0.041919
**GOTERM_BP_DIRECT**	GO:0048663~neuron fate commitment	3	0.041942

**GOTERM_BP_DIRECT**	GO:0048265~response to pain	3	0.041942
**GOTERM_BP_DIRECT**	GO:0008203~cholesterol metabolic process	5	0.042698
**GOTERM_BP_DIRECT**	GO:0007218~neuropeptide signaling pathway	6	0.046422
**GOTERM_BP_DIRECT**	GO:0045653~negative regulation of megakaryocyte differentiation	3	0.046594
**GOTERM_CC_DIRECT**	GO:0045211~postsynaptic membrane	9	0.048761
**GOTERM_CC_DIRECT**	GO:0070062~extracellular exosome	66	0.049893
**KEGG_PATHWAY**	hsa04972:Pancreatic secretion	10	3.33E-05
**KEGG_PATHWAY**	hsa04080:Neuroactive ligand-receptor interaction	14	0.001097
**KEGG_PATHWAY**	hsa04974:Protein digestion and absorption	7	0.004382
**KEGG_PATHWAY**	hsa04260:Cardiac muscle contraction	6	0.010041
**KEGG_PATHWAY**	hsa05034:Alcoholism	9	0.012504
**KEGG_PATHWAY**	hsa00910:Nitrogen metabolism	3	0.035128
**KEGG_PATHWAY**	hsa04726:Serotonergic synapse	6	0.045492
**KEGG_PATHWAY**	hsa05410:Hypertrophic cardiomyopathy (HCM)	5	0.048034

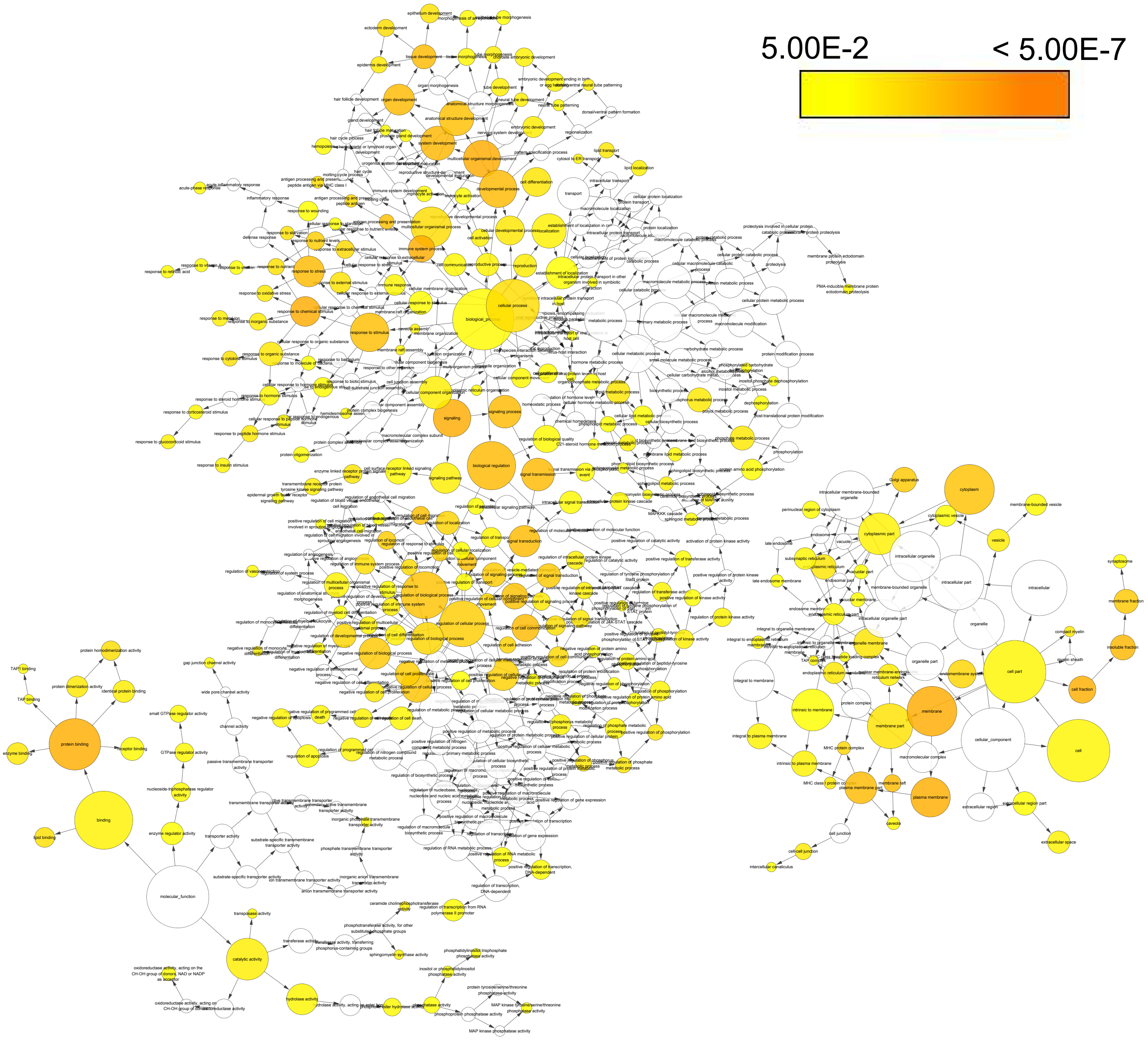

Figure S1. BiNGO enrichment results of AC096751.1 co-expressed genes.

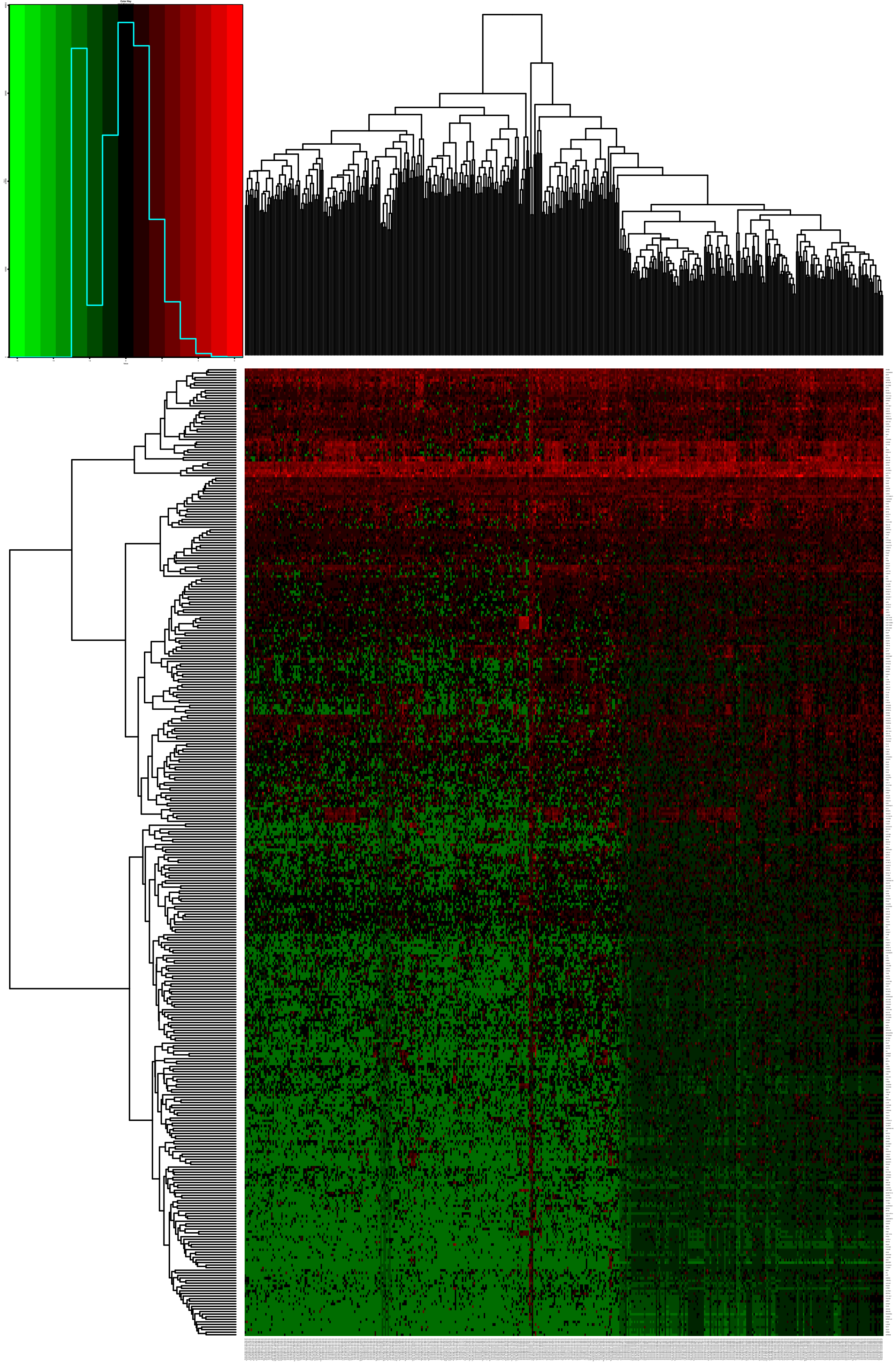

Figure S2. Heat map of the differentially expressed genes between high- and low-AC096751.1 expression phenotypes in COAD.

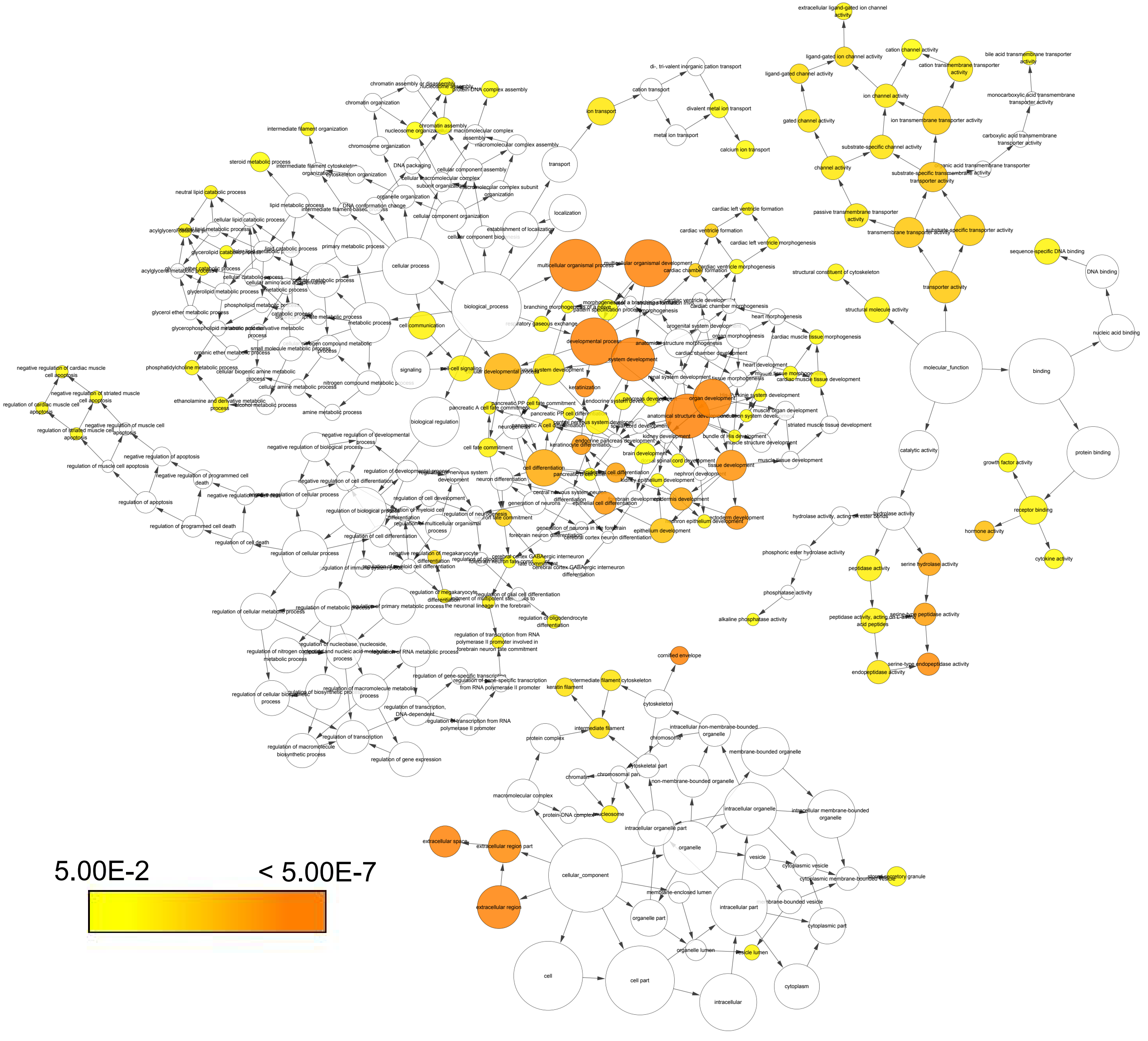

Figure S3. BiNGO enrichment results of DEGs between high- and low-AC096751.1 expression phenotypes in COAD.
